# Dysfunctional neuro-muscular mechanisms explain gradual gait changes in prodromal spastic paraplegia

**DOI:** 10.1186/s12984-023-01206-8

**Published:** 2023-07-15

**Authors:** Christian Lassmann, Winfried Ilg, Tim W. Rattay, Ludger Schöls, Martin Giese, Daniel F. B. Haeufle

**Affiliations:** 1 Multi-level Modeling in Motor Control and Rehabilitation Robotics, Hertie Institute for Clinical Brain Research, University of Tuebingen, Tuebingen, Germany; 2 Section Computational Sensomotorics, Hertie Institute for Clinical Brain Research, University of Tuebingen, Tuebingen, Germany; 3 Department of Computer Engineering, Wilhelm-Schickard-Institute for Computer Science, University of Tuebingen, Tuebingen, Germany; 4 Centre for Integrative Neuroscience (CIN), Tuebingen, Germany; 5 Department of Neurodegenerative Disease, Hertie-Institute for Clinical Brain Research, and Center for Neurology, University of Tuebingen, Tuebingen, Germany; 6 German Center for Neurodegenerative Diseases (DZNE), Tuebingen, Germany; 7 Center for Rare Diseases (ZSE), University of Tuebingen, Tuebingen, Germany; 8 Institute for Modeling and Simulation of Biomechanical Systems, University of Stuttgart, Stuttgart, Germany; 9 Institute of Computer Engineering (ZITI), Heidelberg University, Heidelberg, Germany

**Keywords:** Gait simulation, Spasticity, Hyperreflexia, Prodromal, SPG4, HSP, Movement disorder

## Abstract

**Background:**

In Hereditary Spastic Paraplegia (HSP) type 4 (SPG4) a length-dependent axonal degeneration in the cortico-spinal tract leads to progressing symptoms of hyperreflexia, muscle weakness, and spasticity of lower extremities. Even before the manifestation of spastic gait, in the prodromal phase, axonal degeneration leads to subtle gait changes. These gait changes - depicted by digital gait recording - are related to disease severity in prodromal and early-to-moderate manifest SPG4 participants.

**Methods:**

We hypothesize that dysfunctional neuro-muscular mechanisms such as hyperreflexia and muscle weakness explain these disease severity-related gait changes of prodromal and early-to-moderate manifest SPG4 participants. We test our hypothesis in computer simulation with a neuro-muscular model of human walking. We introduce neuro-muscular dysfunction by gradually increasing sensory-motor reflex sensitivity based on increased velocity feedback and gradually increasing muscle weakness by reducing maximum isometric force.

**Results:**

By increasing hyperreflexia of plantarflexor and dorsiflexor muscles, we found gradual muscular and kinematic changes in neuro-musculoskeletal simulations that are comparable to subtle gait changes found in prodromal SPG4 participants.

**Conclusions:**

Predicting kinematic changes of prodromal and early-to-moderate manifest SPG4 participants by gradual alterations of sensory-motor reflex sensitivity allows us to link gait as a directly accessible performance marker to emerging neuro-muscular changes for early therapeutic interventions.

**Supplementary Information:**

The online version contains supplementary material available at 10.1186/s12984-023-01206-8.

## Background

In many neurodegenerative movement disorders like Parkinson’s disease, cerebellar ataxia, or hereditary spastic paraplegia (HSP), gait impairments are among the leading symptoms. They often appear as the first signs [1–4] and are one of the most disabling features in the progression of these diseases. Recently, it has become possible to quantify specific subtle gait changes in early disease phases or even before the manifestation of clinical disease symptoms (the prodromal phase) [3, 4]. The prodromal phase of movement disorders [5] attracts increasing research interest, as it provides a promising window for early therapeutic intervention before substantially irreversible neurodegeneration has occurred.

We have recently shown for hereditary spastic paraplegia type 4 (SPG4) participants—the most common autosomal dominant and pure motor form of HSP [5, 6]—that specific subtle changes in the kinematic gait pattern can be detected by quantitative movement analysis in the prodromal phase, before the manifestation of spastic gait [7]. Changes that can be observed early are gradually increasing minimum plantarflexion or reducing foot range of motion (RoM) and these changes increase with disease severity [7], leading to gait patterns affecting the ankle, knee, and hip joints [8–10]. Especially the foot RoM and minimum plantarflexion show significant correlations to disease severity already in the prodromal and early manifest stages [7].

On the neuro-muscular level, key pathologies observed in HSP patients are hyperreflexia, leg spasticity, and muscle weakness [11]. The origin of these pathologies is a degeneration of axons in the cortico-spinal tract which mainly affects the long axons responsible for transmitting signals for lower-limb control [12–15]. Due to the length-dependency of the affected axons, early gait changes have been primarily observed in the ankle joint [2, 7]. Brisk patellar and achilles reflexes can be observed in clinical examinations already in the prodromal phase [5]. In the manifest stage, additional spasticity and muscle weakness can be observed in static conditions as well as in gait [9, 16, 17]. However, it is unknown to which part spastic hyperreflexia or muscle weakness contribute to the subtle gait changes observed in the prodromal and early phases.

In order to understand the emerging gait abnormalities in early disease stages, it is crucial to investigate the development on the level of dysfunctional sensory-motor control mechanisms. Forward-dynamic computer simulation with neuro-musculoskeletal models offer the possibility to investigate the effect of isolated sensory-motor alterations [18]. This method allows to reproduce healthy gait [19, 20] and to study the contribution of individual sensory-motor reflexes to gait patterns [18, 21–23]. The effect of the length-dependent axonal degeneration in the cortico-spinal tract, as seen in HSP, on gait can be investigated by gradual manipulation of specific neuro-muscular mechanisms. Incremental bilateral plantarflexor weakness affecting gait was previously investigated by Waterval et al. [24]. Van der Krogt et al. reproduced gait characteristics of children with cerebral palsy by introducing a velocity-dependent stretch reflex, increasing muscle activity for the fast stretch of muscle fibers [25]. Jansen et al. showed how hyperexcitability of muscle spindle reflex loops contribute to hemiparetic gait by investigating length- and velocity feedback [26]. Bruel et al. combined the effects of muscle weakness and hyperreflexia to explain the sensory-motor origin of spastic heel- and toe-walking [27]. In their study, they added muscle spindle-, length-, and force feedback to the two plantarflexor muscles in their model, soleus (SOL) and gastrocnemius medialis (GAS), and introduced muscle weakness by reducing the maximum isometric muscle forces [27]. This shows that gait abnormalities of manifest patients and even gradual gait changes can be explained by sensory-motor alterations in neuro-muscular models of walking.

**Fig. 1:**
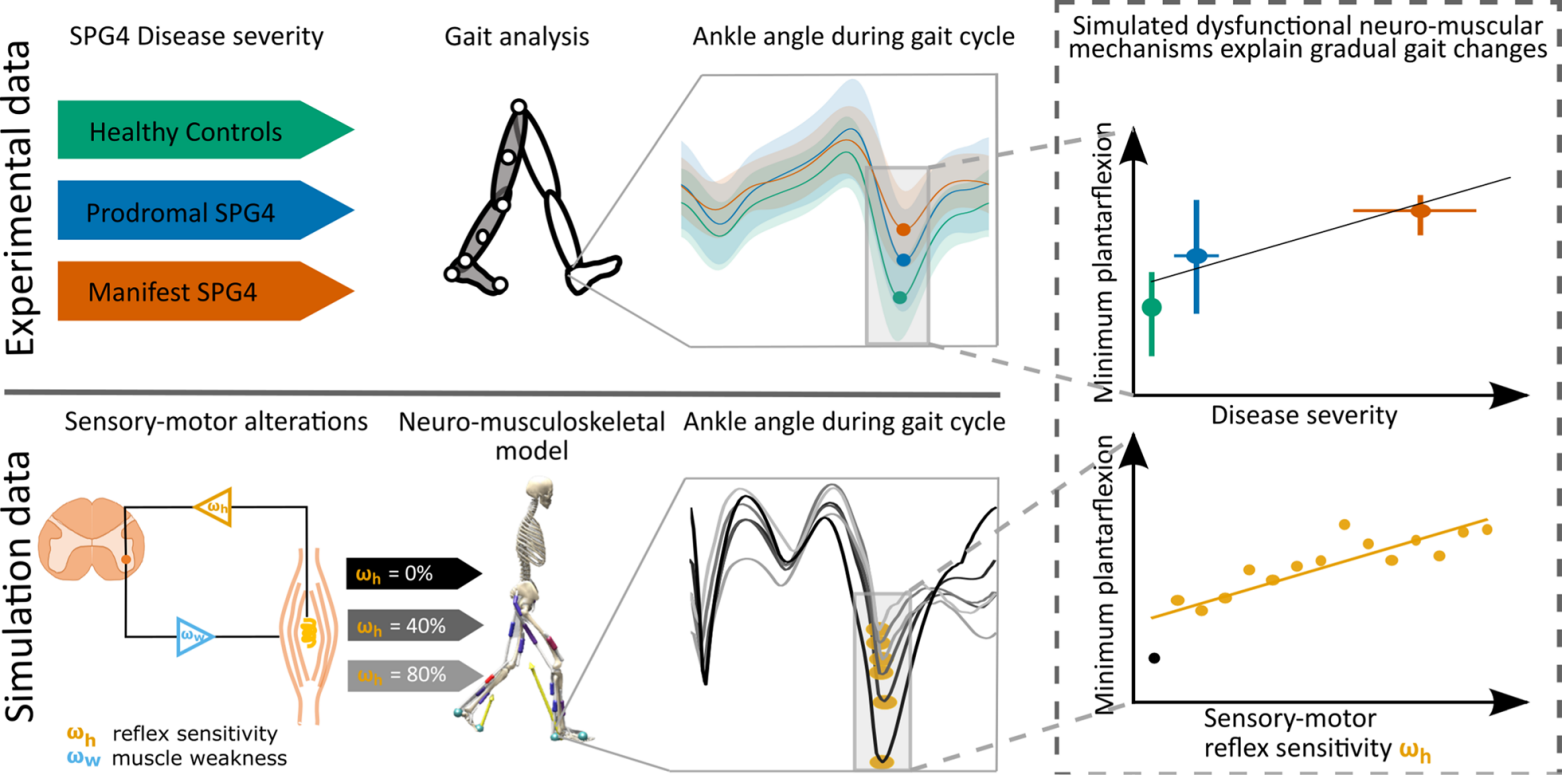
Study Design—Can dysfunctional neuro-muscular mechanisms explain gradual gait changes of prodromal and early-to-moderate manifest SPG4 patients? We first used data of an instrumented gait analysis to investigate gait changes of healthy controls (green), prodromal SPG4 (blue), and manifest SPG4 (red) patients. We identified characteristic changes for the three different groups, which were recently published [7]. Secondly, we introduced and gradually manipulated neuro-muscular mechanisms, i.e. hyperreflexia (muscle spindle velocity feedback, orange), muscle weakness (reduced isometric force, light blue), and their combination in a neuro-musculoskeletal model and expected to predict relative gait changes, as in experimental data

In this study, we hypothesize that a gradual manipulation in sensory-motor reflex sensitivity and muscle weakness can explain the emergence of early gait changes in prodromal participants towards early spastic gait in manifest SPG4 patients (see Fig. 1 for the study design). The gait of prodromal participants and manifest SPG4 patients had an intact gait cycle structure consisting of heel strike, roll-over, push-off and swing phases (here called: heel strike walking). We base our approach on a previously published model predicting healthy human gait kinematics and dynamics [19]. In this model, we gradually manipulate hyperreflexia based on muscle spindle velocity feedback and muscle weakness to determine whether a singular neuro-muscular dysfunction or only their combination can explain the gradual kinematic changes observed in experimental data. We expect that developing gait changes over disease severity of prodromal participants to the spastic gait of mild-to-moderate manifest patients can be predicted by altering plantarflexor and dorsiflexor muscle spindle reflex sensitivity and leg muscle weakness, caused by length-dependent axonal degeneration in SPG4.

## Methods

### Experimental data

We evaluate data from our previously published study [7], which included 17 manifest SPG4 patients, 30 prodromal SPG4, and 23 healthy control participants. Participants were instructed to walk with their typical gait in a self-determined pace. All participants had an intact heel strike walking gait cycle pattern. Participants underwent an instrumented gait analysis in a movement laboratory using an infrared-camera-based motion capture system (VICON FX with ten cameras). Gait cycles were recorded with 41 reflecting markers at a sampling rate of 120 Hz, and extracted by detection of the heel strike event. Trials were smoothed by a Savitzky-Golay polynomial filter and resampled equidistantly to 100 data points per gait cycle. For the analysis, we calculated stride length, gait speed and joint angles, to compare to simulated data.

### Computational model of human gait

We used a neuro-musculoskeletal model to predict kinematics and kinetics of healthy and impaired human gait in forward-dynamic simulations, similar to Bruel et al. [27], implemented in SCONE [31] and HyFyDy [32] (see Additional files 1 and 2). The model is planar (sagittal plane) with seven segments (trunk-pelvis, bilateral thigh, lower-leg, and foot) and seven degrees of freedom (simplified from OpenSim gait2392 [28]). The planar model was used, since the most prominent differences between healthy controls, prodromal SPG4, and manifest SPG4 patients were found in the flexion and extension angles, especially in the foot segment [7]. We modeled seven Hill-type muscles (Millard-equilibrium muscle model [29]), namely gluteus maximus (GLU), iliopsoas (IL), rectus femoris (RECT), vastus intermedius (VAS), gastrocnemius medialis (GAS), soleus (SOL), and tibialis anterior (TA) per leg. Muscle path, optimal fiber length, pennation angle, tendon slack length, and maximum isometric forces were set to the values in the Gait2392 model (see Additional files 1 and 2). Ground contact was modeled using two viscoelastic Hunt-Crossley contact spheres on each foot, serving as heel and toe contacts.

The neuronal control model calculated muscle stimulation signals *U* for each of the fourteen muscles according to a gait-state dependent reflex-based controller (see Table 1) based on [19]. Muscle reflexes were calculated similar to Eq. 1, with the feedback gain and offset parameters being optimized. The controller considered muscle force and length feedback, vestibular feedback, and constant signals to generate muscle excitation. There are positive feedback reflexes self-stimulating the muscle which provides the afferent feedback signal, negative force feedback from SOL to TA, and length feedback from HAM to IL. To stabilize the orientation of the trunk during stance, a proportional-derivative feedback loop was implemented for HAM, IL and GLU. Our addition to the model was a positive self-stimulating muscle spindle velocity feedback (*V+*) to GAS, SOL, and TA to simulate spastic hyperreflexia (see following section, Eq. 1). Reflex gains could differ between five gait phases (early stance, late stance, pre-swing, swing, and late swing), as proposed in previous studies [20, 24, 30]. Reflex delays were set to 5 ms for HAM, IL, and GLU, 10 ms for VAS, and 20 ms for GAS, SOL, and TA, as proposed by Geyer and Herr (2010) [19]. The initial state was selected as proposed by the optimization software SCONE [31]. All files to reproduce the results in SCONE can be found in the (Additional files 1).

### Simulation study design: a model of spastic hyperreflexia and muscle weakness

This study gradually introduced sensory-motor alterations to model healthy, prodromal and early-to-moderate manifest gait in SPG4. We investigated three control scenarios: pure spastic hyperreflexia, pure muscle weakness, and a combination of both. For each of these scenarios, we investigated the magnitude of the respective sensory-motor alterations.

Modeling spastic hyperreflexia and muscle weakness: To model spastic hyperreflexia, we introduced a gain parameter $\omega _h\in [0\%...100\%]$. $\omega _h$ is multiplied by the equation calculating the muscle spindle velocity feedback:

1 $$\begin{aligned} U_V = \omega _h \cdot K_V \cdot (V - V_0) \end{aligned}$$

with *V* and $V_{0}$ being the normalized CE velocity $((L / L_{opt}) / s)$ and the respective constant reference velocity. By increasing $K_V$, we identified $K_V={0.12}{}$ as the maximum velocity feedback gain ($\omega _h = 100\%$) relevant for our study. Higher $K_V$ values led to walking patterns mostly on the toes which therefore did not match the criterion of a heel strike walking pattern anymore. $\omega _h=0\%$ results in a deactivated velocity reflex and $\omega _h=100\%$ results in the maximally investigated velocity reflex sensitivity (hyperreflexia). $\omega _h$ was added to the ankle plantarflexors GAS and SOL, and ankle dorsiflexor TA during the stance and swing phase.

To model muscle weakness, we introduced a gain parameter $\omega _w\in [0\%...100\%]$ which directly reduces the maximum isometric muscle force ($F_{\max }$) of each leg muscle according to the equation:

2 $$\begin{aligned} {\bar{F}}_\text {max}=F_\text {max} - \left( \omega _w * {\bar{F}}_ {\text {manifest}}\right) \end{aligned}$$

$\omega _w=0\%$ represents a model with all muscles at full strength, while $\omega _w=100\%$ represents a reduction of the isometric force by $F_{\text {manifest}}$, i.e. a reduction by 58% of TA, 42% of GAS and SOL, 38% VAS, 35% IL, 11% GLU, and 30% RECT, as reported for manifest HSP patients in Marsden et al. [16].

To model the third scenario, we combined both approaches simultaneously to investigate the interplay of both symptoms. For this, we introduced the parameter $\omega _{hw}$, which sets both, velocity feedback gain $\omega _{h}$ and muscle weakness $\omega _{w}$ simultaneously to $\omega _{h} = \omega _{w} = \omega _{hw}$.

Modeling gradual sensory-motor alterations: To investigate gradual sensory-motor alterations, the magnitude of the gains was increased in 15 steps: $\omega _{h,w,hw}=[0\%, 6.67\%,13.34\%,20\%,...,100\%]$. Low $\omega$-values mean minimal sensory-motor alterations, i.e., low hyperreflexia and muscle weakness, while $\omega$-values of 100% represent the highest alterations investigated in this study. See Fig. 5 for details on the gradual change of velocity feedback gain and muscle weakness and their combination.

### Optimization of controller parameters

For each of the scenarios described above, all other controller parameters were optimized. These are the feedback gains of the other reflexes (length, force, and vestibular) within each state (Table 1) and the initial joint angles. We used the open-source software SCONE with Hyfydy for the optimization, a dedicated software to run and optimize predictive neuro-muscular simulations [31, 32]. The cost function for the optimization

3 $$\begin{aligned} J_{\text {cost}} = 100 * J_{\text {gait}} + 0.1 * J_{\text {effort}} + 0.1 * J_{\text {ankle joint}} + 0.01 * J_{\text {knee joint}} + 10 * J_{\text {grf}} \end{aligned}$$

and the weights were adopted from the pre-defined settings in SCONE [31]. Similar weights were used in previous studies to predict unimpaired gait [24, 30]. Veerkamp et al. stated the importance of adding ground reaction forces to this cost function [33]. The cost function considered

4 $$\begin{aligned} J_{\text {gait}}={\left\{ \begin{array}{ll} 1, &{} \text {if COM height }< 0.85 * \text { initial COM height}\\ 1 - {\bar{v}} &{} \text {if } {\bar{v}} < 1 \text { (slower than } v_{\min } \text {)}\\ 0, &{} \text {else} \end{array}\right. } \end{aligned}$$

the difference between the normalized average gait speed

5 $$\begin{aligned} {\bar{v}} = \frac{1}{n} \sum _{\text {step}=1}^{n} \frac{v_{\text {step}}}{v_{\min }} \end{aligned}$$

with $v_{\text {step}}$ being the average velocity in each step, and a minimally desired average gait speed of $v_{\min }=1 \frac{m}{s}$ representing an average gait speed of mild-to-moderate SPG4 participants [7]. It further considers an effort measure from [34] minimizing metabolic energy expenditure of muscles ($J_{\text {effort}}$), a joint measure penalizing hyperextension and -flexion of the ankle (Eq. 6) and knee (Eq. 7) joints, and ground reaction force measure penalizing peak vertical forces during gait over a certain threshold ($J_{\text {grf}}$):

6 $$\begin{aligned}{} & {} J_{\text {ankle joint}}={\left\{ \begin{array}{ll} 0, &{} \text {if }-60^{\circ }<\text { ankle angle }< 60^{\circ } \\ (|\text {ankle angle}| - 60)^{2}, &{} \text {else} \end{array}\right. } \end{aligned}$$

7 $$\begin{aligned}{} & {} J_{\text {knee joint}}={\left\{ \begin{array}{ll} 0, &{} \text {if knee angle moment }> -5\text {Nm} \\ |\text {knee angle moment}|, &{} \text {else} \end{array}\right. } \end{aligned}$$

8 $$\begin{aligned}{} & {} J_{\text {grf}}={\left\{ \begin{array}{ll} |\frac{\text {peak GRF}}{\text {Body weight}}| - 1.5* \text {Body weight}, &{} \text {if peak GRF }> 1.5* \text {Body weight and time }> 1s \\ 0, &{} \text {else} \end{array}\right. } \end{aligned}$$

SCONE uses the Covariance Matrix Adaptation Evolutionary Strategy from Igel et al. [35]. Initial joint angles and control parameters were the free parameters in the optimization. All optimizations started from the same initial state, which was selected as proposed by the optimization software SCONE. The stochastic component of the optimization algorithm was initialized with a random seed. Translation states and coordinate velocities at t=0 were fixed throughout the entire optimization and evaluation. The optimization was stopped when the average reduction of the cost function score was less than 0.0001% compared to the previous iteration. We simulated gait for 30 s, always starting from the same initial parameters. We only considered simulations walking without falling until the simulation ended (t=30 s). The heel strike event was detected when the vertical component of the ground reaction force was greater than a threshold (initially 0.1*BodyWeight, later optimized by SCONE). To ensure periodicity, we excluded the initial gait cycle from the evaluation. To make this approach reproducible, we added all files necessary to reproduce the simulations as additional files to the (Additional file 1 including the parameter files for the initial parameters).

Finally, we resampled the remaining cycles to 100 data points and calculated the average across all gait cycles.

### Data evaluation

We compared the simulation output to the experimental data for specific relevant gait features identified in a previous study [7]. As gradually altering features in prodromal and manifest SPG4, they identified the minimum plantarflexion, the foot range of motion (RoM), and the maximum ground clearance of the heel. For manifest SPG4 the knee angle at heel strike increased and the maximum heel angle and knee RoM reduced significantly. Furthermore, gait speed and stride length were reduced over disease progression for manifest SPG4 patients [7]. Hip, knee, and ankle joint angle kinematics during the gait cycle were compared between healthy controls, prodromal SPG4 participants, and manifest SPG4 patients. We compared the simulation results to nine previously experimentally identified key features depicting group and progression changes [7]: (1) ankle RoM, (2) minimum plantarflexion (swing phase), (3) ankle angle at heel strike, (4) ankle angle at maximum heel ground clearance, (5) knee RoM, (6) maximum knee angle, (7) knee angle at heel strike, (8) gait speed, and (9) stride length. Peak and average muscle activation for SOL, GAS, and TA was calculated for each gait phase (early stance, late stance, pre-swing, swing, and late swing). SOL and TA co-activation values were calculated with average muscle activation values for each of the five gait phases:

9 $$\begin{aligned} CA_\text {phase} = {\left\{ \begin{array}{ll} \frac{sol + ta}{2} * \frac{sol}{ta}, &{} \text {if }sol< ta \\ \frac{ta + sol}{2} * \frac{ta}{sol}, &{} \text {if }ta < sol \end{array}\right. } \end{aligned}$$

where *sol* and *ta* represent the mean muscle activation for a certain gait phase. For statistical comparison Kruskal-Wallis test and post hoc Dunn’s test for multiple group comparisons were used. We report statistical significance as **$p<0.0056$ (Bonferroni corrected with 9 feature comparisons), and ***$p<0.001$.

We used the SPRS score to categorize participants into clinical disease severity and find possible explanations by increasing velocity feedback gains in the simulations [36].

Spearman’s *rho* was used to identify significant correlations of increased muscle spindle velocity feedback and increased muscle weakness for the nine gait features and optimization parameters, e.g., force feedback gains of individual muscles and metabolic energy expenditure [34].

## Results

### Experimental gait data

As recently published, instrumental gait analysis revealed significant group differences between healthy controls (HC), prodromal SPG4 participants, and manifest SPG4 patients [7]. In this study, we compare their experimental data to our simulations’ outcomes and therefore include their results. All participants performed a self-determined heel strike walking. They extracted joint angle kinematics and other gait parameters, as described in detail by Lassmann et al. [7].

In their study, several gait parameters showed significant differences between manifest SPG4 participants, prodromal SPG4 participants and healthy controls with increasing effects in manifest SPG4 patients. Minimum plantarflexion ($$p<0.001^{***}$$) and ankle angle at maximum heel ground clearance ($$p<0.001^{***}$$) were significantly higher for healthy controls in comparison to manifest SPG4 participants. In contrast, ankle angle features of prodromal SPG4 participants did not differ significantly from manifest SPG4 participants. Spearman’s *rho* showed a gradual increase of these features with disease severity ($$rho=0.48$$, $$p<0.001^{***}$$; $$rho=0.5$$, $$p<0.001^{***}$$, respectively). For prodromal SPG4 participants, the knee RoM ($$p=0.0014^{**}$$) was significantly increased and the knee angle at heel strike ($$p=0.0016^{**}$$) was significantly reduced in comparison to manifest SPG4 patients. The gait speed and stride length were increased for healthy controls, but not for prodromal SPG4 participants. Table 3 shows mean values and standard deviation for all nine analyzed features of the three groups.

**Fig. 2:**
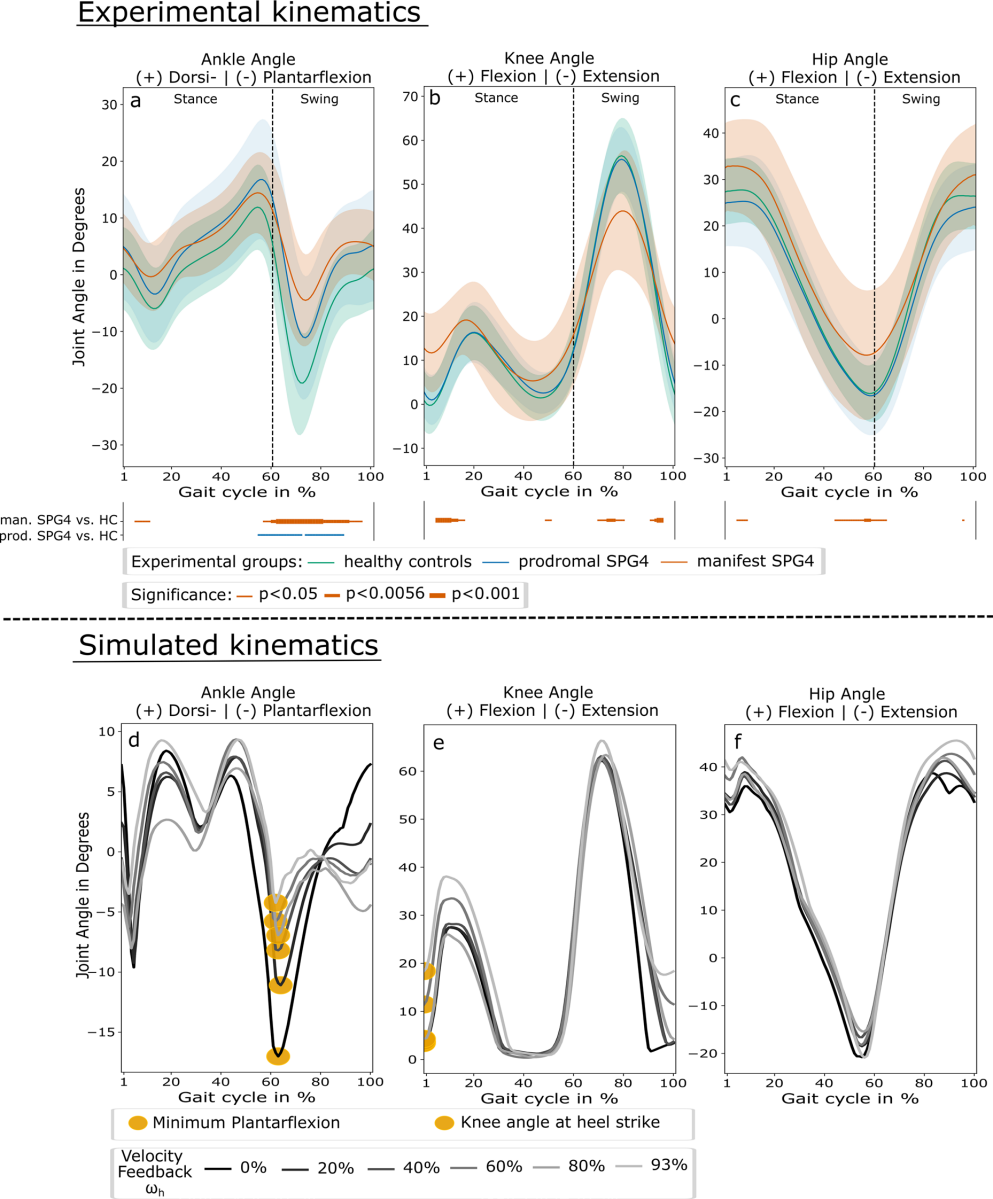
Experimental and simulated joint angle kinematics over gait cycle. **a**–**c** mean flexion and extension angles of ankle, knee, and hip joints over the gait cycle in percent for healthy controls (green), prodromal SPG4 (blue), and manifest SPG4 (red), with their standard deviation. Significant periods are indicated as lines above the trajectory plots indicating different levels of significance (thin line: $p<0.05$, intermediate line: $p<0.0056$, and bold line: $p<0.001$. Differences between prodromal SPG4 vs. HC and manifest SPG4 vs. HC are shown as blue and red lines, respectively. **d**–**f**: flexion and extension angles of ankle, knee, and hip joints over the gait cycle in percent for different levels of velocity feedback gains (color coded from black: $\omega _h=0\%$, light grey: $\omega _h=93\%$) of plantarflexor and dorsiflexor muscles. The extracted features are highlighted yellow, namely the minimum plantarflexion **d** and knee angle at heel strike (**e**)

Kinematics of the ankle, knee, and hip joint during the gait cycle showed differences between HC (green), prodromal SPG4 (blue), and manifest SPG4 (red in Fig. 2*a-c*). The most prominent differences occurred during the swing phase, e.g., the increasing minimum plantarflexion angle from healthy controls to prodromal participants and manifest SPG4 patients (Fig. 2*a* at around 70% of the gait cycle), indicating a progression with disease severity. Furthermore, the increased knee angle at heel strike in the manifest group is visible (Fig. 2*b*, the beginning of the gait cycle).

### Neuro-musculoskeletal gait model

#### Simulated healthy walking pattern

The simulation of the not adapted controller [19] can generate a gait pattern which shows high similarity to healthy human gait. Figure 2*d-f* in black ($\omega = 0\%$) and Table 2 show the results for the model with optimized controller parameters (optimized in Scone). We found reduced maximum ankle dorsiflexion and a more extended swing phase compared to our experimental data.

#### Effect of increasing velocity feedback gain

**Fig. 3:**
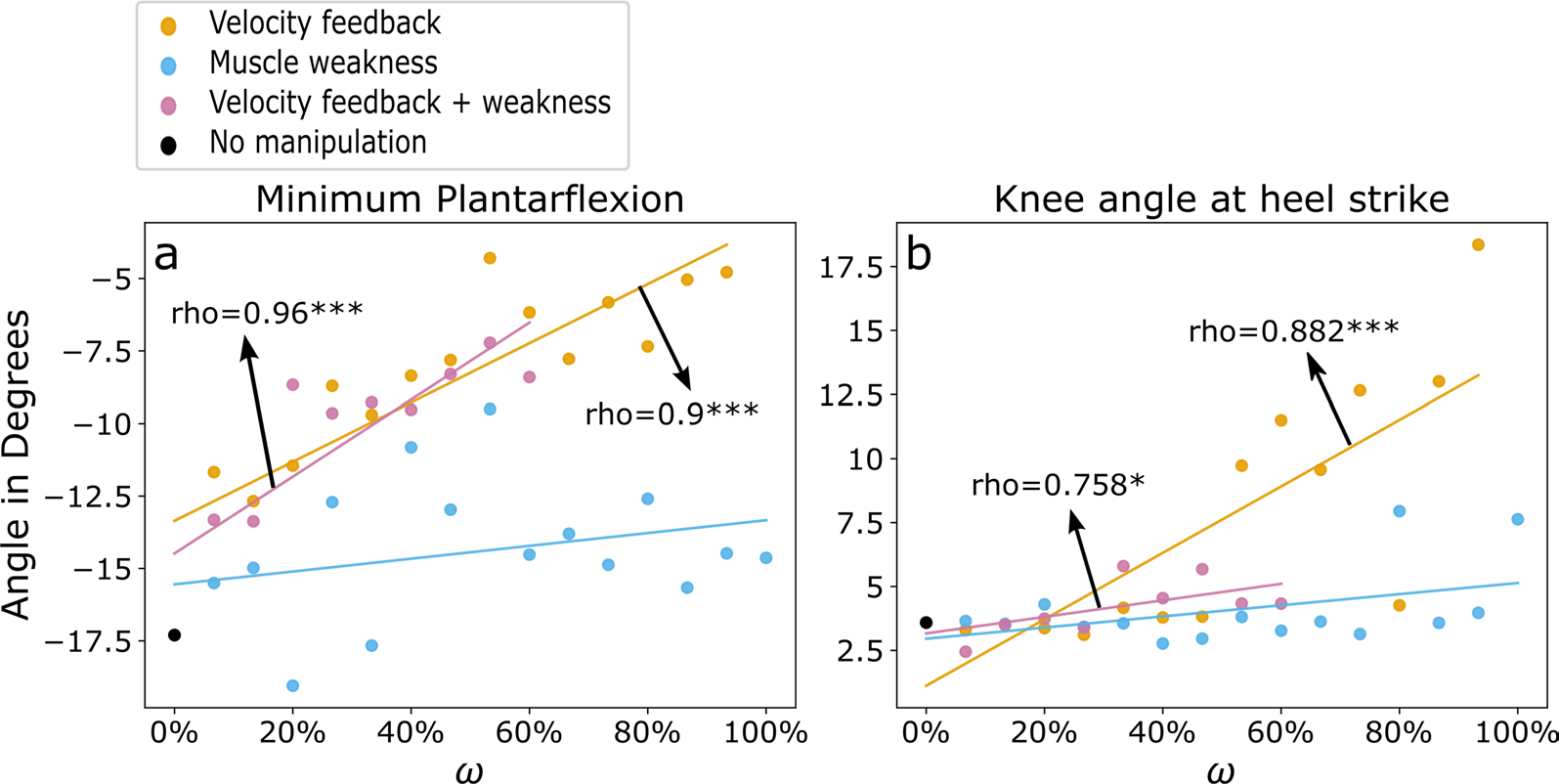
Progression of minimum plantarflexion and knee angle at heel strike for simulation szenarios. Increasing levels of velocity feedback gain (orange), muscle weakness (light blue), and velocity feedback gain + muscle weakness (purple) with simulation iteration $$\omega =0\%$$ to $$\omega = 100\%$$ and linear fits. a) minimum plantarflexion and b) knee angle at heel strike are shown with significant spearman correlation coefficients. Asterisks indicate significant levels of: $$^{**}$$: $$p<0.0056$$, and $$^{***}$$: $$p<0.001$$. For $$\omega _h=100\%$$ and $$\omega _{hw}>60\%$$ optimization led to no stable walking simulations

With increasing levels of velocity feedback gain $$K_V$$ ($$\omega _h$$) to plantarflexor and dorsiflexor muscles during the stance and swing phase, several kinematic changes occurred within heel strike walking. Ankle: The minimum plantarflexion angle reduced from $$-$$17.3$$^\circ$$ ($$\omega _h = 0\%$$) to $$-$$11.4$$^\circ$$ at $$\omega _h = 20\%$$ and further to $$-$$8.35$$^\circ$$ ($$\omega _h = 40\%$$) and $$-$$4.8$$^\circ$$ ($$\omega _h = 93\%$$) (see Fig. 2*d*). This resulted in a strong correlation between increasing velocity feedback gains and minimum plantarflexion ($$rho=0.9$$, $$p<0.001^{***}$$, compare Fig. 3*a*). In addition, also the ankle angle at heel strike was gradually increased ($$rho=-0.87$$, $$p<0.001^{***}$$). Knee: At heel strike, the knee angle was gradually increased from $$\omega _h \ge 53\%$$ to $$\omega _h = 93\%$$ ($$rho=0.88$$, $$p<0.001^{***}$$, compare Fig. 2*e* and Fig. 3*b*). For comparison with experimental data, the results of different iterations of increasing velocity feedback gain are shown in Table 2 and all results with correlations in Table 4.

**Fig. 4:**
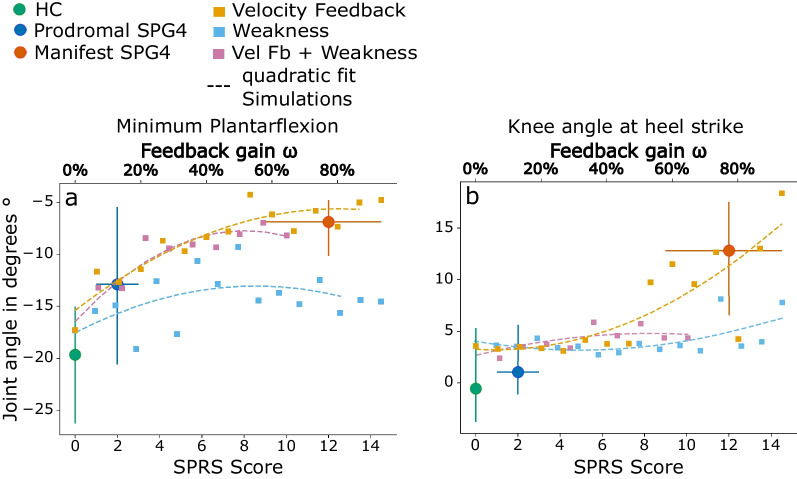
Gait features over disease severity and simulated dysfunctional neuro-muscular mechanisms. **a** Minimum plantarflexion and **b** knee angle at heel strike of experimental data and simulations over disease severity (SPRS score) and velocity feedback gain $\omega _h$, muscle weakness $\omega _w$, and velocity feedback gain and muscle weakness $\omega _{hw}$. The three experimental groups are color-coded with healthy controls (green), prodromal SPG4 (blue), and manifest SPG4 (red). Shown are averaged values for SPRS scores as blue and red circles. Error bars are showing distributions of all groups with their mean SPRS score (position on lower x-axis) and standard deviation of SPRS score indicated by horizontal error bars. Orange, light blue, and purple squares are showing simulation data at different gains of velocity feedback ($\omega _h$), muscle weakness ($\omega _w$), and velocity feedback gain and muscle weakness ($\omega _{hw}$), respectively (upper x-axis). Quadratic fits for simulations are shown in the respective color

**Table 1:** The controller is based on Geyer and Herr [19] and has several components and states: constant actuation (C), muscle reflex, and pelvis tilt proportional-derivative (PD) primitives

	Early Stance	Late Stance	Pre-swing	Swing	Late swing
Eevent initiating gait state	GRF greater than threshold	Sagittal distance stance foot	Contralateral foot enters early stance	GRF lower than threshold	Sagittal distance swing foot
GLU	C, PD+	C, PD+	C	F+	F+
HAM	C, PD+	C, PD+		F+	F+
IL	C, PD-	C, PD-	C	PD+, L+/-(HAM)	PD+, L+/-(HAM)
VAS	C, F+	C, F+			
GAS	F+, *V+*	F+, *V+*	F+, *V+*	*V+*	*V+*
SOL	F+, *V+*	F+, *V+*	F+, *V+*	*V+*	*V+*
TA	L+, F-(SOL), *V+*	L+, F-(SOL), *V+*	L+, F-(SOL), *V+*	L+, F-(SOL), *V+*	L+, F-(SOL), *V+*

Muscle reflex primitives are based on normalised muscle length (L) or force (F) feedback. In this study, we added positive velocity feedback (*V+*) to simulate hyperreflexia. Positive feedback (L+, F+, and *V+*) directly stimulates a muscle based on its on proprioceptive signals. Furthermore, negative force feedback from SOL may inhibit TA (F-(SOL)) and length feedback from HAM may inhibit IL (L±(HAM)). The active controller components depend on the five gait states: early stance, late-stance, pre-swing, swing (S) and late swing (state machine) and the states are switched when the specified events are detecte (second row of the table)

**Table 2:** Mean results for minimum plantarflexion and knee angle at heel strike with standard deviation (STD) for experimental (upper three rows) and simulated data

Gait feature	Minimum plantarflexion	Knee angle at heel strike
HC	– 20.8 ± 9.5***	0.8 ± 6.81***
Prod SPG4	– 13.4 ± 10.5	2.8 ± 5.7**
Man SPG4	– 8.5 ± 7.9	11.9 ± 7.9
$\omega =0\%$	– 17.3	2
$\omega _h=20\%$	– 11.4	1.9
$\omega _h=40\%$	– 8.4	3.8
$\omega _h=60\%$	– 6.2	11.5
$\omega _h=80\%$	– 7.3	4.3
$\omega _h=100\%$	n.a.	n.a.
$\omega _w=20\%$	– 19.05	4.3
$\omega _w=40\%$	– 10.82	2.77
$\omega _w=60\%$	– 14.52	3.27
$\omega _w=80\%$	– 12.59	7.95
$\omega _w=100\%$	– 14.63	7.63
$\omega _{hw}=20\%$	– 8.67	3.74
$\omega _{hw}=40\%$	– 9.52	4.55
$\omega _{hw}=60\%$	– 8.4	4.33
$\omega _{hw}=80\%$	n.a.	n.a.
$\omega _{hw}=100\%$	n.a.	n.a.

Asterisks indicate significance: $$^{**}$$ if $$p<0.0056$$ (Bonferroni corrected with 9 feature comparisons), and $$^{***}$$ if $$p<0.001$$ in comparison to manifest SPG4 participants. Different levels of the velocity feedback gain ($$\omega _h$$), weakness ($$\omega _w$$), and combined velocity feedback and weakness ($$\omega _{hw}$$) are given for comparison

**Fig. 6:**
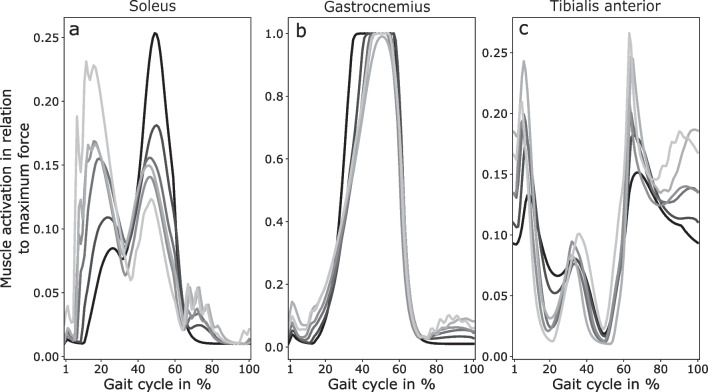
Simulated muscle activation for velocity feedback gains. Muscle activation of soleus, gastrocnemius and tibialis anterior over the gait cycle in percent for different levels of velocity feedback gain

SOL average activation was increased during the early stance phase ($$rho=0.95$$, $$p<0.001^{***}$$) and reduced during lift-off ($$rho=-0.75$$, $$p=0.0012^{**}$$) Fig. 6*a*. During landing, there was a greater SOL activation ($$rho=0.84$$, $$p<0.001^{***}$$). For GAS, the average activation during the early stance phase was increased with increasing $$\omega _h$$, showing a prolonged GAS activation over the stance phase; however, with a shortened peak muscle activation period Fig. 6*b*. During the landing phase, the GAS average activation increased with higher velocity feedback gain ($$rho=0.97$$, $$p<0.001^{***}$$). TA peak activation increased at early stance ($$rho=0.87$$, $$p<0.001^{***}$$) Fig. 6*c*. During landing, TA activity increased with $$\omega _h$$ ($$rho=0.94$$, $$p<0.001^{***}$$) (see Fig. 6*c*). SOL-TA co-activation increased during early stance ($$rho=0.87$$, $$p<0.001^{***}$$) and landing ($$rho=0.93$$, $$p<0.001^{***}$$) with increasing $$\omega _h$$.

All iterations with increasing muscle spindle velocity feedback gain, except for $\omega _h=100\%$, could be optimized to a stable walking simulation.

#### Effect of increasing muscle weakness

**Fig. 7:**
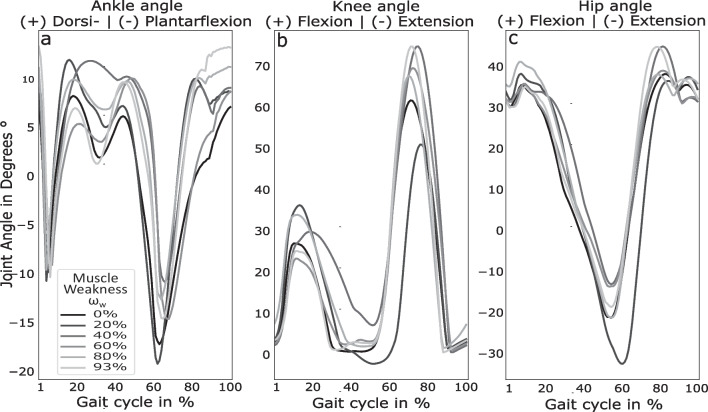
Simulated kinematics with increasing muscle weakness. Flexion and extension angles of the ankle, knee, and hip joints over the gait cycle in percent for different levels of muscle weakness. For more details on different gait features see Table 5

The gradual increase of muscle weakness $$F_{\text {max}}$$ ($$\omega _w$$) as reported by Marsden et al. [16] resulted in an increased ankle angle at heel strike ($$rho=0.8$$, $$p<0.001^{***}$$, compare Fig. 3a). The maximum knee angle differed between simulation scenarios in a range of 52$$^\circ$$ ($$\omega _w = 20\%$$) and 75$$^\circ$$ ($$\omega _w = 66.7\%$$), with no significant correlation over increased muscle weakness. Other investigated features did not show a specific pattern with increasing muscle weakness. All simulations with increasing muscle weakness ($$\omega _w = 0\%... 100\%$$) could be optimized to a stable heel strike walking simulation. For all simulation results, see Table 5 and Fig. 7.

#### Combined velocity feedback gain and muscle weakness

**Fig. 8:**
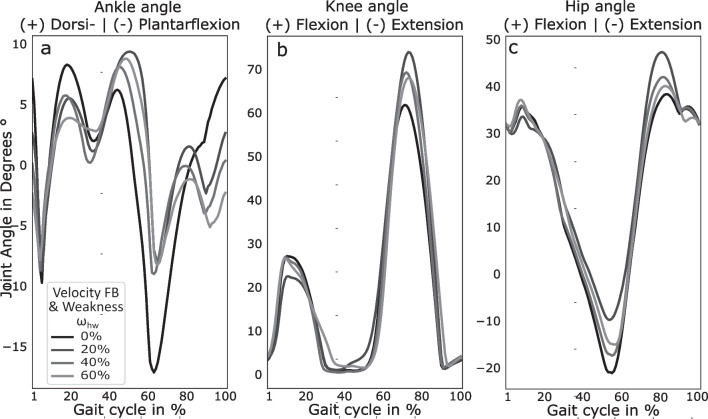
Simulated kinematics with increasing hyperreflexia and muscle weakness. Flexion and extension angles of the ankle, knee, and hip joints over the gait cycle in percent for velocity feedback gain and muscle weakness. For more details on different gait features see Table 6

**Fig. 9:**
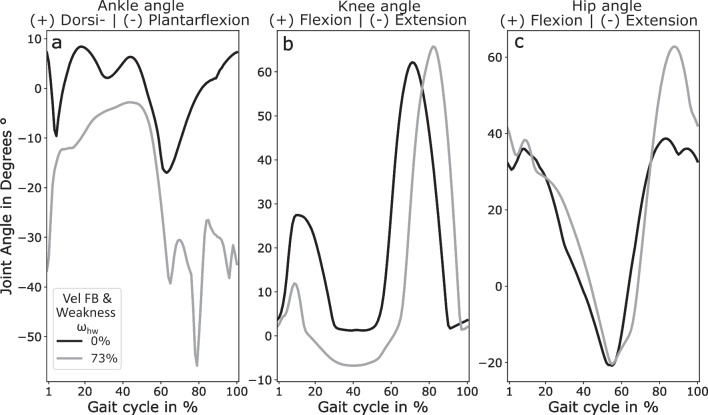
Simulated kinematics for toe-gait. Flexion and extension angles of ankle, knee, and hip joints over the gait cycle in percent for velocity feedback gain and muscle weakness of $\omega _{hw}=73\%$ and $\omega _{hw}=0\%$ for comparison. For this scenario, the optimization process produced a controller with stable toe gait with initial ball contact. For more details on different gait features see Table 6

The combination of a gradual increase of velocity feedback gain and muscle weakness ($$\omega _{hw}$$) resulted in patterns similar to the velocity feedback gain scenario. During the swing phase, the minimum plantarflexion angle was reduced for higher $$\omega _{hw}$$ ($$rho=0.96$$, $$p<0.001^{***}$$, see Fig. 3*a*). The ankle angle reduced at heel strike ($$rho=-0.96$$, $$p<0.001^{***}$$) and increased at maximum heel ground clearance ($$rho=0.98$$, $$p<0.001^{***}$$). Gait speed and stride length were reduced to comparable levels as in the velocity feedback gain scenario, however, with no significant correlation to increased $$\omega _{hw}$$ (see Table 6 and Fig. 8). The optimizer failed to produce stable heel strike walking with $$\omega _{hw} \ge 60\%$$, showing a reinforced effect by combining the gradually increased velocity feedback gain and muscle weakness. At $$\omega _{hw} = 73\%$$ the optimization dismissed the heel strike walking but produced a stable toe-walking pattern with initial ball contact, increased hip flexion angle and a time offset at maximum knee flexion angle (compare Fig. 9).

#### Dysfunctional neuro-muscular mechanisms and disease severity

In the experimental data, the minimum plantarflexion and the knee angle at heel strike show gradual changes over disease severity (compare Fig. 2a-c). In Fig. 4 and Table 2 these two gait features are shown over disease severity in comparison to the simulation results over $\omega _{h,w,hw}$. The iterative decrease of the minimum plantarflexion by increasing $\omega _h$ and $\omega _{hw}$ can be fitted to the gradual decrease over disease severity in SPG4 participants (see Fig. 4a). Muscle weakness alone shows no distinct connection to disease severity. For the knee angle at heel strike, manifest SPG4 patients differ significantly from HC and prodromal SPG4 participants (Table 2). The knee angle at heel strike with velocity feedback gains $\omega _h\ge 53.3\%$ is comparable to the manifest SPG4 patients, whereas a lower velocity feedback gain leads to knee angles comparable to prodromal SPG4 participants (see Fig. 4b). $\omega _w$ and $\omega _{hw}$ in our simulations can not explain the increase in the knee angle at heel strike.

#### Optimized control parameters and cost terms

**Fig. 10:**
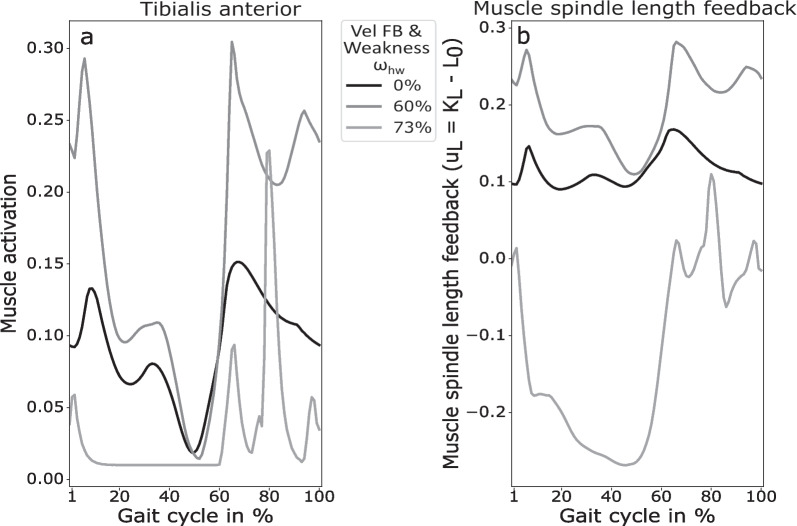
Tibialis anterior activation and muscle spindle length feedback. TA activation and muscle spindle length feedback over the gait cycle in percent for different levels of combined velocity feedback and muscle weakness ($\omega _{hw}=0\%$, no alteration; $\omega _{hw}=60\%$ heel strike gait; and $\omega _{hw}=73\%$ toe-gait). For $\omega _{hw}=73\%$ the muscle spindle length offset ($L_0$) is increased in comparison to other scenarios, which reduces TA activation and reciprocal inhibition of plantarflexor muscles

For each specified velocity feedback gain and/or muscle weakness parameters ($$\omega _h$$, $$\omega _w$$, or $$\omega _{hw}$$), we optimized all other controller parameters to find a suitable gait minimizing our locomotion cost function (Eq. 3). This re-optimization resulted in changes in the cost terms and the controller parameters and reflected the possibility of the rest of the nervous system adapting to specific sensory-motor changes. For increasing velocity feedback gain the cost term $$J_{\text {effort}}$$ metabolic energy expenditure [34] increased with increasing $$\omega$$ ($$\omega _h: rho=0.79$$, $$p<0.001^{***}$$). The controller parameter *length feedback gain* of TA (optimized over the whole gait cycle) increased with higher velocity feedback gains ($$rho=0.72$$, $$p=0.002^{**}$$). The *force feedback gains* of SOL and GAS during lift-off and swing phases decreased with higher velocity feedback gains ($$rho=-0.94$$, $$p<0.001^{***}$$, for both) and combined velocity feedback and muscle weakness ($$rho_{\text {SOL}}=-0.98$$, $$p_{\text {SOL}}<0.001^{***}$$; $$rho_{\text {GAS}}=-0.99$$, $$p_{\text {GAS}}<0.001^{***}$$). For the combined controller of muscle weakness and velocity feedback gain, the offset of TA muscle spindle length feedback ($$L_0$$) was optimized to an increased value of 1.07 (as a fraction of the optimal TA muscle fiber length of 9.8cm) for the toe-gait scenario ($$\omega _{hw}=73\%$$), in comparison: for all other combined controller scenarios ($$L_0(\omega _{hw} \in [6.67\%...60\%]) = 0.65 \pm 0.003$$). This offset leads to a reduced TA activation during stance, lift-off, and landing (Fig. 10). For more details on the optimized parameters, see Table 7.

## Discussion

We hypothesized, that the subtle gait changes in heel strike walking observed in prodromal SPG4 participants could be explained by gradual changes in neuro-muscular feedback mechanisms. To investigate this, we implemented gradually increased sensitivity of sensory-motor reflex in a neuro-musculoskeletal forward simulation of heel strike walking [19]. Increasing levels of velocity feedback gain in plantarflexor and dorsiflexor muscles resulted in kinematic and muscular changes comparable to those observed in prodromal participants and early-to-moderate manifest SPG4 patients.

### Increasing hyperreflexia explains the development of early gait changes in SPG4

On the kinematic level, the earliest gait changes in prodromal SPG4 participants occur in the foot segment and ankle joint [7]. Increasing muscle spindle velocity feedback ($\omega _h$) in the simulation caused several gait changes that are in line with kinematic changes of heel strike walking in prodromal participants and early-to-moderate manifest SPG4 patients.

In the simulation, the minimum plantarflexion increased gradually with $\omega _h$ ($rho=0.9$, $p<0.001$) to comparable levels as it increased over disease severity, measured by the SPRS score [36], in the experimental data of prodromal and early-to-moderate manifest SPG4 participants ($rho=0.49$, $p<0.001$). With $\omega _h \ge 53\%$ the minimum plantarflexion saturates, as it has been shown in [7] for early-to-moderate manifest SPG4 patients.

The ankle RoM was identified as key feature of kinematic changes in prodromal and manifest SPG4 participants [7] and used to cluster manifest HSP patients into severity-related groups [2]. In the simulation, the ankle RoM reduced gradually with increasing $\omega _h$ ($rho=-0.99$, $p<0.001$), as in the experimental data with disease severity ($rho=-0.5$, $p<0.001$). However, the absolute values did not fit the experimental data due to reduced maximum dorsiflexion in all simulations.

Comparable to the experimental data with disease severity ($rho=0.48$, $p<0.001$), the knee angle at heel strike was gradually increased with greater velocity feedback gain ($rho=0.88$, $p<0.001$). For low velocity feedback gains ($\omega _h < 53\%$), the knee angle at heel strike remained on a constant level comparable to healthy controls and prodromal SPG4 participants. With greater velocity feedback gains, the knee angle at heel strike increased, matching the kinematic changes in manifest SPG4 patients.

Currently, there is no measurement or biomarker linking our velocity feedback gain parameter $\omega _h$ to disease severity. However, when plotting kinematic features like minimum plantarflexion and knee angle at heel strike of experimental data over SPRS score, which indicates disease severity [36], and of simulated data over sensory-motor reflex sensitivity $\omega _h$, the plot suggests reproducing the gradually changing gait features of prodromal and early-to-moderate manifest SPG4 participants with disease severity (see Fig. 4). These findings allow us to conclude that velocity-dependent hyperreflexia can explain the development of earliest gait changes in prodromal participants and early spastic gait in patients with hereditary spastic paraplegia type 4 and shows the importance of gait as directly accessible performance marker for early therapeutic interventions.

### Increasing hyperreflexia predicts changes in muscular coordination

The increasing velocity feedback gain $\omega _h$ has consequences beyond the kinematic changes. Optimizing all other neuronal control parameters for any given $\omega _h$, increased SOL and TA activity during the early stance and swing phase, with a higher level of co-activation. Rinaldi et al. reported a similarly increased co-activation of antagonist ankle muscles (SOL-TA) during the stance and swing phase in manifest HSP patients [17]. Martino et al. found a prolonged activation of ankle plantarflexor muscles in manifest HSP, which could be replicated in our simulated GAS activation, however, with a shorter peak period [9].

Our simulations’ metabolic energy expenditure [34] was positively correlated with increasing velocity feedback gains. This result is in line with Rinaldi et al. who report an increase in energetic consumption in manifest HSP patients [17].

These findings indicate that the increased velocity feedback gain, a model representation of hyperreflexia in the ankle joint muscles, predicts not only kinematic but also muscular and energetic trends observed in prodromal and early-to-moderate manifest SPG4 patients.

### Severely spastic gait in manifest SPG4

In contrast to other simulation studies that focus on severe manifest spastic gait with altered gait patterns, we investigated the prodromal and early phases of spastic gait with an intact gait cycle structure consisting of heel strike, roll-over, push-off, and swing phases (here called: heel strike walking).

We did not find the kinematic changes occurring in prodromal and early-to-moderate manifest SPG4 participants for increasing muscle weakness. However, the combined effects of hyperreflexia and muscle weakness $\omega _{hw}$ show the importance of muscle weakness in manifest hereditary spastic paraplegia. By simultaneously increasing both velocity feedback gain and muscle weakness, we found a toe-gait pattern (Fig. 9), which is characteristic of later manifest stages of hereditary spastic paraplegia. Our results suggest a decrease of TA activation in the toe-gait scenario, resulting in a decrease of TA-SOL reciprocal inhibition. In combination with the increased plantarflexor velocity feedback gain, this leads to an over-activity of plantarflexor muscles during the stance and swing phase.

Previous simulation studies investigated severe manifest gait in different movement disorders by introducing hyperreflexia and muscle weakness in similar optimization approaches [24, 27, 30]. As in our study, all of these studies used a reflex controller based on Geyer and Herr [19] and optimized control parameters by the CMA-ES algorithm [35]. Bruel et al. also added more complex reflex circuits to SOL, GAS, and TA muscles [27]. Cost functions for optimizing control parameters differ slightly between all studies: All studies included a cost term regularizing gait speed and falling (similar to Eq. 4) and a cost term for metabolic energy expenditure. Waterval et al. and Bruel et al. penalized joint hyperextension and -flexion in a similar way as done in our study, by considering knee ligament moments (Eq. 7) and ankle angles exceeding certain levels (Eq. 6) [24, 27]. In addition, we penalized high ground reaction forces (Eq. 8).

Waterval et al. simulated bilateral plantarflexor weakness by incrementally introducing GAS and SOL muscle weakness [24]. They report that gait altered meaningfully when maximum isometric muscle force was reduced to less than 40%. Ong et al. (2019) reduced maximum isometric force of SOL and GAS to 25% (mild), 12.5% (moderate), and 6.25% (severe) to simulate plantarflexor weakness [30]. They found that gait changes mainly occur in their moderate and severe scenarios and lead to heel-walking kinematics. In Bruel et al. only parts of their heel-walking criteria were fulfilled when reducing maximum isometric force of SOL and GAS. In our study, we reduced muscle force to levels found by Marsden et al. [16], with a minimum muscle force of 58% remaining in plantarflexors. We found no exclusive effect of the investigated muscle weakness on pathological gait in SPG4 patients, which might be explained by the still remaining isometric force of more than 40%. Bruel et al. showed that increased velocity- and force-related sensory-motor reflexes of GAS and SOL lead to pathological toe-walking patterns, which can be seen in later stages of manifest spastic patients [27]. In another simulation study, Jansen et al. used hyper-excitability of muscle spindle length- and velocity reflex loops to simulate hemiparetic gait in a neuro-musculoskeletal model [26]. They found that both feedback mechanisms introduced to SOL, GAS, Vastus (VAS), and Rectus femoris (RECT), can lead to specific gait impairments, such as reduction of ankle dorsiflexion and decreased knee flexion during stance.

### Study limitations

In the combined sensory-motor reflex scenario of increased velocity feedback gain and muscle weakness, we assumed a simultaneous linear development of both factors from 0% to 100%. The experimental results of Rattay et al. suggest that lower leg spasticity and muscle weakness emerge contiguously, but later than hyperreflexia [5], which was found in almost all prodromal SPG4 participants [5, 7]. For higher $\omega _{hw}$ in the combined scenario, several optimizations did not find a stable walking gait. Further investigations in the longitudinal development of muscle weakness and hyperexcitability of muscle spindle reflex loops in SPG4 patients are necessary to understand the interplay of these symptoms.

All objectives of the cost function for the parameter optimization influence the resulting gait pattern. Therefore, it is necessary to mention that our model is only one possible explanation of neuro-muscular mechanisms that may lead to the experimentally observed gait changes in SPG4 participants. For our simulations, we used a combined cost function that penalized excessive ground reaction forces, as suggested by Veerkamp et al. [33]. Furthermore, the minimum gait speed was set to 1 m/s, which is the average gait speed of the early-to-moderate SPG4 group in Laßmann et al. [7]. Hyper-extension and -flexion of ankle and knee joints were penalized to ensure normal gait patterns. We introduced this cost function since we were interested in the subtle gait changes of prodromal and early-to-moderate SPG4 participants, who still perform a heel strike walking pattern. To simulate more severe stages of SPG4, a different cost function may be needed, to allow a less constrained gait pattern as previously also suggested by Bruel et al. [27]. Therefore, this study could be extended by exploring how cost functions might change over disease severity. Specifically, as also noted by Bruel et al. [27], the excessive GAS activation during stance (see Fig. 6) may affect the simulation outcome of e.g. the ankle angle kinematics and could be regularized to a more realistic muscle activation pattern.

The length-dependent axonal degeneration in the cortico-spinal tract of SPG4 patients [13] suggests that spinal reflex changes may emerge first for distal reflex loops. For this reason, we studied gradual velocity feedback gains only at the most distal muscles (GAS, SOL, and TA). Also Martino et al. found altered muscle activation in the most distal muscles [9]. For muscle weakness, we considered an affection of all simulated muscles, as reported in Marsden et al. [16]. Altering the sensory-motor reflex sensitivity in more proximal muscles may increase the simulation prediction accuracy of kinematic changes also in the other joints – at the cost of interpretation complexity. Nevertheless, it is crucial to investigate further the impact of muscle activation and hyperexcitability of the knee and hip muscle reflex loops, e.g., as Di Russo et al. did to investigate the effect of different sensory-motor reflex sensitivities on gait speed and stride length [37].

The model we used is limited to simulating walking in the sagittal plane (two-dimensional). In severe manifest SPG4 patients, hip adductor spasticity is a common symptom [38] and leads to instability. Simulating the 3D gait pattern of SPG4 patients would be needed to include a more detailed symptomatic pattern of muscle spasticity and weakness. Furthermore, the controller we used considers a set of muscle reflexes that does not necessarily represent muscle reflexes in human walking correctly, e.g. Bruel et al. considered a more complex reflex circuit model for SOL, GAS, and TA muscles [27].

### Conclusion and outlook

Very early kinematic changes in the gait pattern present a directly accessible performance measure for prodromal and manifest SPG4 participants [7]. Here we identified sensory-motor reflex sensitivity changes as a possible explanation for these subtle kinematic changes. In our model, the gradual increase of reflex sensitivity can explain the gradual change in heel strike walking observed with increasing disease severity. On the other hand, muscle weakness could be compensated by other adapting spinal reflexes and did not lead to the observed kinematic changes. From this, we speculate that early pharmacological interventions to reduce spasticity (e.g., by baclofen) might reduce subtle gait changes by reducing the sensory-motor reflex sensitivity. However, the side-effects of increased muscle weakness may be compensated intraindividual through adapting spinal reflexes. This thought experiment indicates that pharmacological reduction of spasticity in early SPG4 patients could delay the onset of manifest spastic gait. In the currently running longitudinal experimental study, we will further investigate individual kinematic changes over time and simulate the development of sensory-motor reflex alterations to link gait changes to neuro-muscular mechanisms for future therapeutic interventions [5, 7]. Further studies are needed to objectively measure altered sensory-motor reflex loops and axonal damage in prodromal and early-to-moderate SPG4 participants, e.g., a dynamometer-based H-reflex measure and corticomuscular coherence measure, respectively.

### Supplementary Information

**Additional file 1.** The neuro-musculoskeletal model, the controller, initial parameters, the cost function and a batch-file running all simulations in SCONE with Hyfydy are attached as additional material.

**Additional file 2.** Video files showing simulated gait can be found as additional material.

**Additional file 3.** Additional figures and tables.

## Figures and Tables

**Fig. 5:**
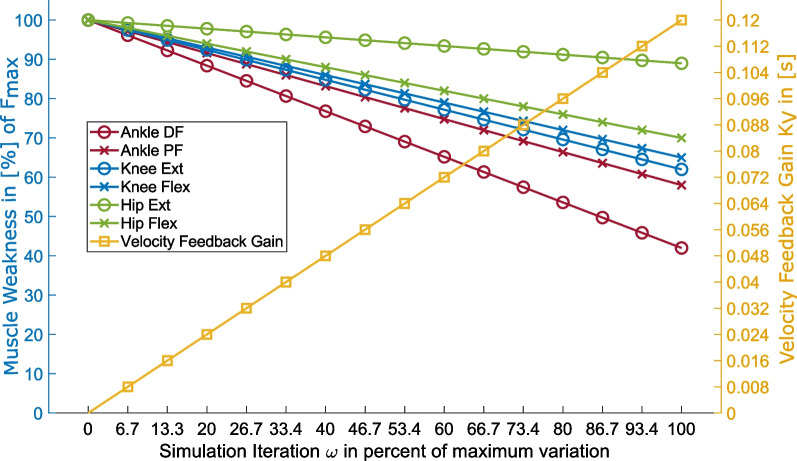
Corresponding values of muscle weakness and velocity feedback gain for simulation iterations $\omega$. Velocity feedback gains for SOL, GAS and TA and percentage of maximum isometric forces for plantarflexors, dorsiflexors, knee, and hip flexors and extensors, with their relative change in $\omega$. *DF* ≡ Dorsiflexion, *PF* ≡ Plantarflexion, *Ext* ≡ Extension, and *Flex* ≡ Flexion

## Data Availability

The datasets for this manuscript are not publicly available because raw data regarding human participants (e.g., genetic raw data, personal data) are not shared freely to protect the privacy of the human participants involved in this study; no consent for open sharing has been obtained. Requests to access an anonymous data set and simulation data should be directed to Christian Lassmann.

## Appendix

See Figs. 5, 6, 7, 8, 9, 10 and Tables 3, 4, 5, 6, 7.

### Experimental results from our previous study

**Table 3:** Mean results for gait features with standard deviation (STD) for experimental data taken from Laßmann et al. [7]

Gait feature	HC	Prod SPG4	Man SPG4
Ankle RoM	33.7 ± 8.5***	31.5 ± 8.3	25.2 ± 6.9
Min Plantarflexion	– 20.8 ± 9.5***	– 13.4 ± 10.5	– 8.5 ± 7.9
Ankle at HS	1.1 ± 7.2	5 ± 9.3	4.7 ± 6.3
Ankle at max HGC	– 18.6 ± 8.7***	– 11.3 ± 10.6	– 6.7 ± 8.5
Knee RoM	60.2 ± 4.9***	58.2 ± 6.3**	47.1 ± 11.3
Knee max angle	57.7 ± 7.9	57 ± 7.2	47 ± 12.6
Knee at HS	0.8 ± 6.8***	2.8 ± 5.7**	11.9 ± 7.9
Gait speed [m/s]	1.36 ± 0.1***	1.28 ± 0.1	1.09 ± 0.2
Stride length [cm]	146 ± 90	137 ± 11	116 ± 19

*RoM* ≡ Range of Motion, *HS* ≡ Heel strike, *HGC* ≡ Heel ground clearance. Asterisks indicate significance: ** if $p<0.0056$ and *** if $p<0.001$ in comparison to manifest SPG4 participants

### Increasing velocity feedback gain

**Table 4:** Kinematic features for velocity feedback gain results over all iterations and correlation

Velocity Feedback $\omega _h$	Gait Speed	Stride Length	Ankle RoM	Min PF	Ankle at HS	Ankle at max HGC	Knee RoM	Knee max angle	Knee at HS
0%	1.20	1.48	25.43	– 17.30	7.24	– 14.70	60.99	62.27	3.59
6.7%	1.05	1.31	19.74	– 11.67	3.99	– 9.66	65.00	66.42	3.30
13.3%	1.10	1.36	19.60	– 12.67	3.83	– 8.95	63.13	64.98	3.50
20%	1.16	1.44	19.00	– 11.45	2.31	– 8.05	61.69	63.02	3.37
26.7%	1.07	1.35	16.25	– 8.70	0.95	– 5.75	62.29	63.34	3.11
33.3%	1.10	1.43	16.99	– 9.70	– 0.02	– 7.38	62.72	62.88	4.17
40%	1.05	1.35	16.22	– 8.35	– 0.51	– 5.11	61.92	63.36	3.79
46.7%	1.03	1.33	15.77	– 7.81	– 1.35	– 3.93	62.94	64.12	3.82
53.3%	1.05	1.34	15.93	– 4.29	– 0.74	– 2.24	64.35	65.51	9.73
60%	1.08	1.39	15.69	– 6.17	– 1.10	– 3.27	62.22	62.70	11.50
66.7%	1.08	1.39	15.30	– 7.78	– 2.29	– 3.97	62.79	63.44	9.57
73.3%	1.04	1.34	14.86	– 5.82	– 1.65	– 1.98	60.87	63.10	12.66
80%	1.03	1.32	15.09	– 7.35	– 4.42	– 3.52	62.97	63.95	4.26
86.7%	1.05	1.38	14.12	– 5.04	– 1.67	– 2.32	61.03	61.73	13.01
93%	1.06	1.33	13.72	– 4.78	– 0.73	– 2.32	65.52	67.12	18.35
Correlation Iteration 0– 14	$$rho=-0.53$$ $$p=0.04$$	$$rho=-0.32$$ $$p=0.25$$	$$rho=-0.99$$ $$p<0.001^{***}$$	$$rho=0.9$$ $$p<0.001^{***}$$	$$rho=-0.87$$ $$p<0.001^{***}$$	$$rho=0.88$$ $$p<0.001^{***}$$	$$rho=0.06$$ $$p=0.82$$	$$rho=0.06$$ $$p=0.84$$	$$rho=0.88$$ $$p=0.001^{***}$$

Shown are all walking simulations for gradual increasing velocity feedback gains. In the bottom row spearman correlation coefficients for the respective features are shown. *RoM* ≡ Range of Motion, *PF* ≡ Plantarflexion, *HS* ≡ Heel strike, and *HGC* ≡ Heel ground clearance

### Increasing muscle weakness

**Table 5:** Kinematic features for muscle weakness results over all iterations and correlation

Weakness $\omega _w$	Gait Speed	Stride Length	Ankle RoM	Min PF	Ankle at HS	Ankle at max HGC	Knee RoM	Knee max angle	Knee at HS
0%	1.20	1.48	25.43	– 17.30	7.24	– 14.70	60.99	62.27	3.59
6.7%	1.18	1.45	26.80	– 15.50	8.58	– 12.55	66.13	67.85	3.65
13.3%	1.10	1.35	24.89	– 14.98	9.30	– 10.24	68.55	69.89	3.54
20%	1.07	1.54	31.11	– 19.05	8.83	– 4.98	53.30	52.06	4.30
26.7%	0.96	1.18	21.00	– 12.71	4.89	– 9.04	72.13	73.37	3.42
33.3%	1.13	1.42	26.78	– 17.66	8.32	– 14.76	66.48	67.88	3.56
40%	1.14	1.33	22.84	– 10.82	9.24	– 3.64	74.37	75.43	2.77
46.7%	0.98	1.18	22.44	– 12.97	8.49	– 9.73	73.02	74.22	2.96
53.3%	0.96	1.45	28.33	– 9.50	11.66	2.46	61.01	61.93	3.81
60%	1.10	1.33	24.72	– 14.52	8.92	– 12.91	68.46	70.41	3.27
66.7%	1.08	1.32	24.38	– 13.80	10.63	– 10.50	74.27	75.48	3.63
73.3%	1.07	1.28	25.85	– 14.87	10.63	– 12.78	72.90	74.42	3.14
80%	1.14	1.50	23.94	– 12.59	11.34	– 9.08	66.08	68.18	7.95
86.7%	0.99	1.30	26.83	– 15.66	11.19	– 12.50	68.20	69.68	3.58
93.3%	1.11	1.42	27.84	– 14.48	13.42	– 9.36	74.30	75.43	3.97
100%	1.11	1.51	28.65	– 14.63	12.16	– 11.72	65.65	67.87	7.63
Correlation Iteration 0 – 15	$$rho=-0.19$$ $$p=0.47$$	$$rho=-0.09$$ $$p=0.74$$	$$rho=0.19$$ $$p=0.48$$	$$rho=0.33$$ $$p=0.213$$	$$rho=0.8$$ $$p<0.001^{***}$$	$$rho=0.06$$ $$p=0.81$$	$$rho= 0.25$$ $$p=0.35$$	$$rho=0.36$$ $$p=0.18$$	$$rho=0.26$$ $$p=0.33$$

Shown are all walking simulations for gradual increasing muscle weakness. In the bottom row spearman correlation coefficients for the respective features are shown. *RoM* ≡ Range of Motion, *PF* ≡ Plantarflexion, *HS* ≡ Heel strike, and *HGC* ≡ Heel ground clearance

### Increasing velocity feedback gain and muscle weakness

**Table 6:** Kinematic features for velocity feedback gain and muscle weakness results over all iterations and correlation

Velocity FB + Weakness $\omega _{hw}$	Gait speed	Stride length	Ankle RoM	Min PF	Ankle at HS	Ankle at max HGC	Knee RoM	Knee max angle	Knee at HS
0%	1.20	1.48	25.43	– 17.30	7.24	– 14.70	60.99	62.27	3.59
6.7%	1.21	1.40	20.03	– 13.32	1.59	– 8.47	64.36	66.82	2.45
13.3%	1.06	1.33	21.45	– 13.37	2.87	– 10.72	63.23	66.52	3.50
20%	0.96	1.20	18.23	– 8.67	2.56	– 2.98	72.93	74.56	3.74
26.7%	1.12	1.36	18.60	– 9.64	0.04	– 5.96	65.40	67.29	3.40
33.3%	1.05	1.42	17.61	– 9.27	3.45	– 5.44	62.23	63.39	5.80
40%	1.03	1.33	17.26	– 9.52	0.25	– 3.89	68.82	69.94	4.55
46.7%	1.07	1.40	17.38	– 8.30	0.10	– 5.45	64.83	65.70	5.68
53.3%	1.06	1.30	16.74	– 7.22	2.09	– 1.84	73.45	74.70	4.34
60%	0.98	1.26	17.11	– 8.40	– 2.21	– 4.88	66.54	69.26	4.33
66.7%	–	–	–	–	–	–	–	–	–
73.3%	0.96	1.46	56.11	– 70.33	– 36.15	– 31.53	72.69	66.19	2.06
Correlation Iteration 0–9	$$rho=-0.52$$ $$p=0.13$$	$$rho=-0.26$$ $$p=0.47$$	$$rho=-0.98$$ $$p<0.001^{***}$$	$$rho=0.96$$ $$p<0.001^{***}$$	$$rho=-0.96$$ $$p<0.001^{***}$$	$$rho=0.98$$ $$p<0.001^{***}$$	$$rho=0.12$$ $$p=0.75$$	$$rho=0.01$$ $$p=0.99$$	$$rho=0.76$$ $$p=0.011$$

Shown are all walking simulations for gradual increasing velocity feedback gains and muscle weakness. In the bottom row spearman correlation coefficients for the respective features are shown. *RoM* ≡ Range of Motion, *PF* ≡ Plantarflexion, *HS* ≡ Heel strike, and *HGC* ≡ Heel ground clearance

### Optimized parameters

**Table 7:** Correlation of optimization parameters for each simulation experiment

Condition	Optimization	Effort	TA[$K_L$]	TA$[L_0$]	TA-SOL[$K_F$]	SOL[$K_F$]	GAS[$K_F$]	VAS[$K_F$]	VAS[$C_0$]
VelFB	$$rho=0.78$$, $$p=0.001^{***}$$	$$rho=0.79$$, $$p<0.001^{***}$$	$$rho=0.72$$, $$p=0.002^{**}$$	$$rho=0.49$$, $$p=0.064$$	$$rho=-0.03$$, $$p=0.919$$	$$rho=-0.94$$, $$p<0.001^{***}$$	$$rho=-0.94$$, $$p<0.001^{***}$$	$$rho=-0.44$$, $$p=0.098$$	$$rho=-0.5$$, $$p=0.056$$
Weakness	$rho=-0.02$, $p=0.931$	$rho=-0.02$, $p=0.931$	$rho=0.41$, $p=0.113$	$rho=-0.01$, $p=0.957$	$rho=0.27$, $p=0.311$	$rho=0.54$, $p=0.031$	$rho=-0.23$, $p=0.399$	$rho=0.34$, $p=0.2$	$rho=0.0$, $p=1.0$
VelFB + Weakness	$$rho=0.63$$, $$p=0.05$$	$$rho=0.66$$, $$p=0.038$$	$$rho=0.47$$, $$p=0.174$$	$$rho=0.39$$, $$p=0.26$$	$$rho=0.48$$, $$p=0.162$$	$$rho=-0.98$$, $$p<0.001^{***}$$	$$rho=-0.99$$, $$p<0.001^{***}$$	$$rho=-0.48$$, $$p=0.162$$	$$rho=-0.84$$, $$p=0.002^{**}$$

Shown are spearman correlation coefficients for optimization parameters for each simulation experiment. *TA * ≡ tibialis anterior, *SOL * ≡ soleus, *GAS* ≡ gastrocnemius medialis, *VAS* ≡ vastus intermedius, *KL* ≡ length feedback gain, *L0* ≡ length offset, *KF * ≡ force feedback gain, and *C0* ≡ constant

## Abbreviations

HSP: Hereditary Spastic Paraplegia
SPG4: Hereditary Spastic Paraplegia type 4
RoM: Range of motion
SOL: Soleus
GAS: Gastrocnemius medialis
IL: Iliopsoas
RECT: Rectus femoris
VAS: Vastus intermedius
TA: Tibialis anterior
