# Supplementary material for: Dysfunctional neuro-muscular mechanisms explain gradual gait changes in prodromal spastic paraplegia
Source: J Neuroeng Rehabil. 2023 Jul 15;20:90. doi: 10.1186/s12984-023-01206-8 (PMC10349428; doi:10.1186/s12984-023-01206-8)
Supplement: Supplementary file 3 — Additional file 3. Additional figures and tables. [file 12984_2023_1206_MOESM3_ESM.pdf]

Additional file 3

Additional figures

Figure 5. Corresponding values of muscle weakness and velocity feedback gain for simulation iterations ω.

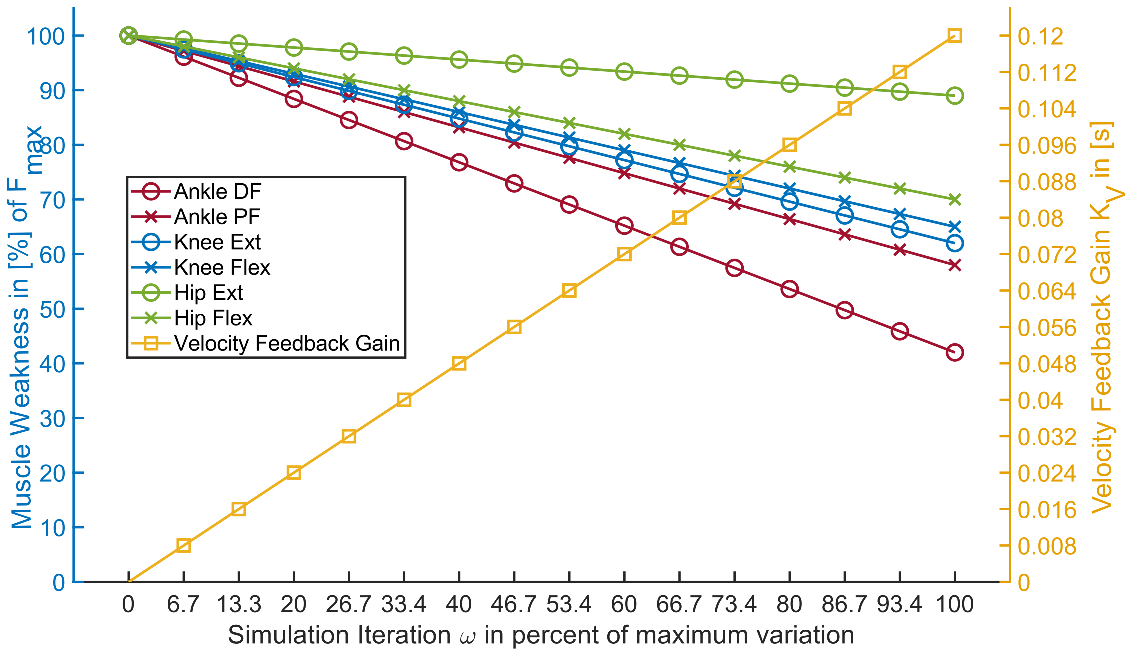

Figure 5: Velocity feedback gains for SOL, GAS and TA and percentage of maximum isometric forces for plantarflexors, dorsiflexors, knee, and hip flexors and extensors, with their relative change in ω. DF ≡ Dorsiflexion, PF ≡ Plantarflexion, Ext ≡ Extension, and Flex ≡ Flexion.

Figure 6. Simulated muscle activations for velocity feedback gains

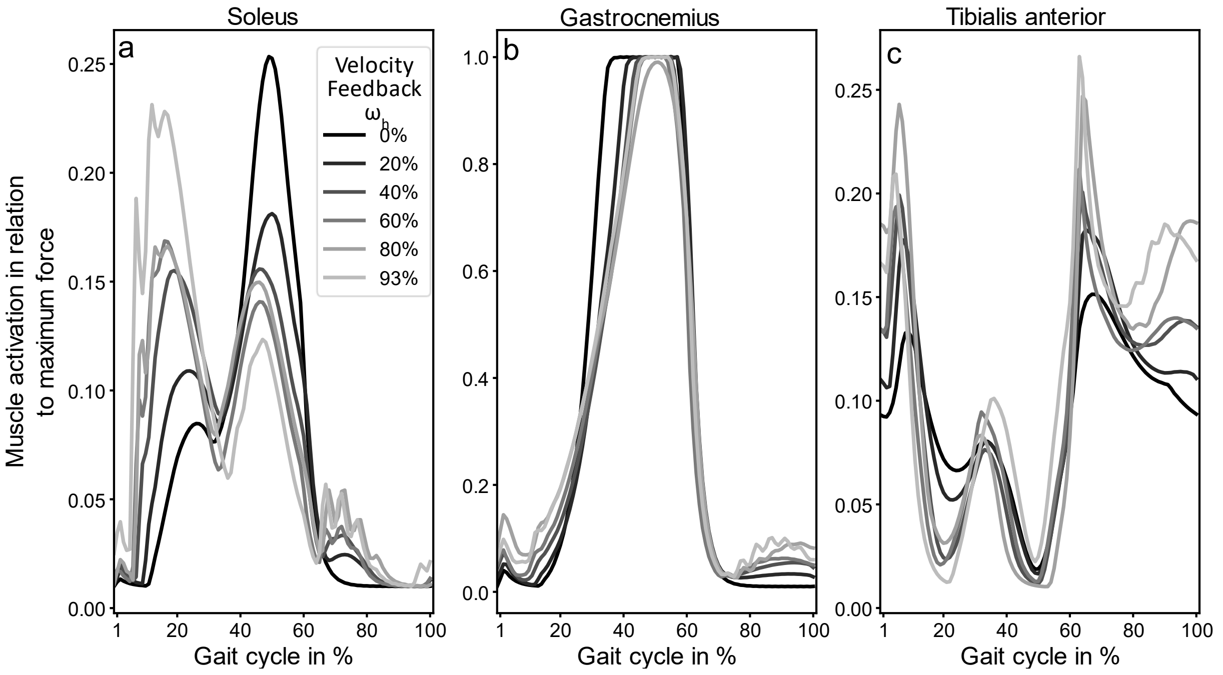

Figure 6: Muscle activation of Soleus, Gastrocnemius and Tibialis anterior over the gait cycle in percent for different levels of velocity feedback.

Figure 7. Simulated kinematics with increasing muscle weakness.

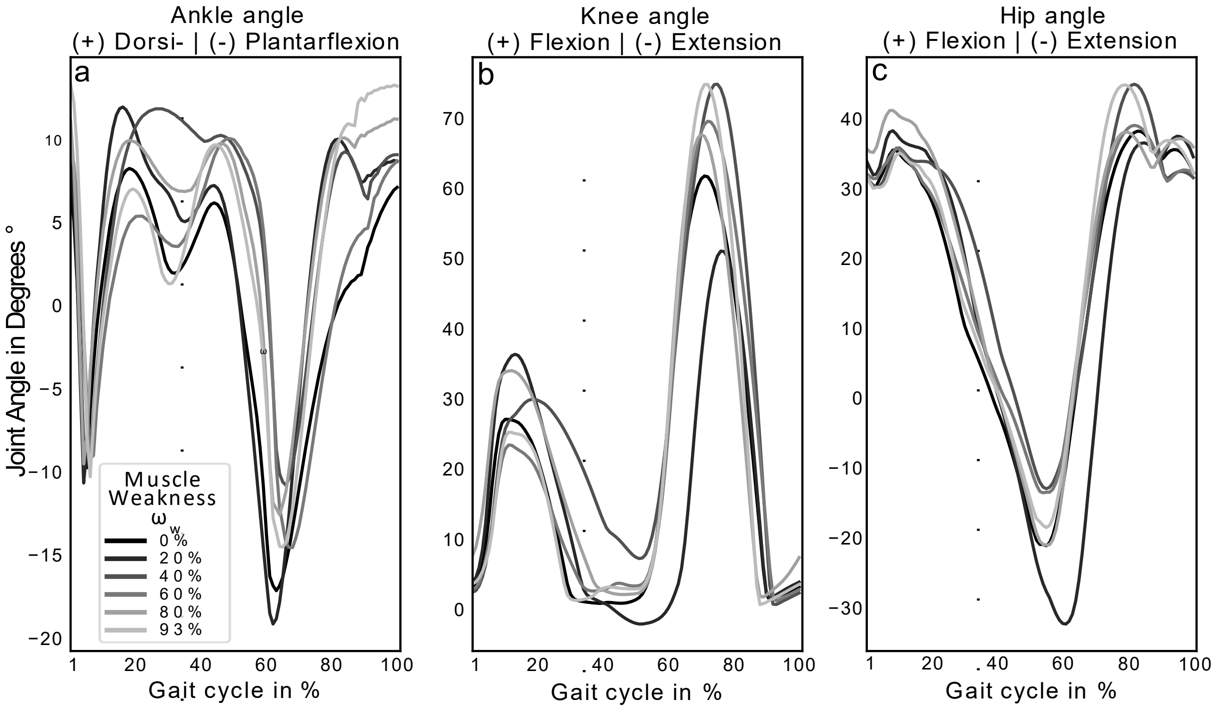

Figure 7: Flexion and extension angles of ankle, knee, and hip joints over the gait cycle in percent for different levels of muscle weakness.

Figure 8. Simulated kinematics with increasing hyperreflexia and muscle weakness.

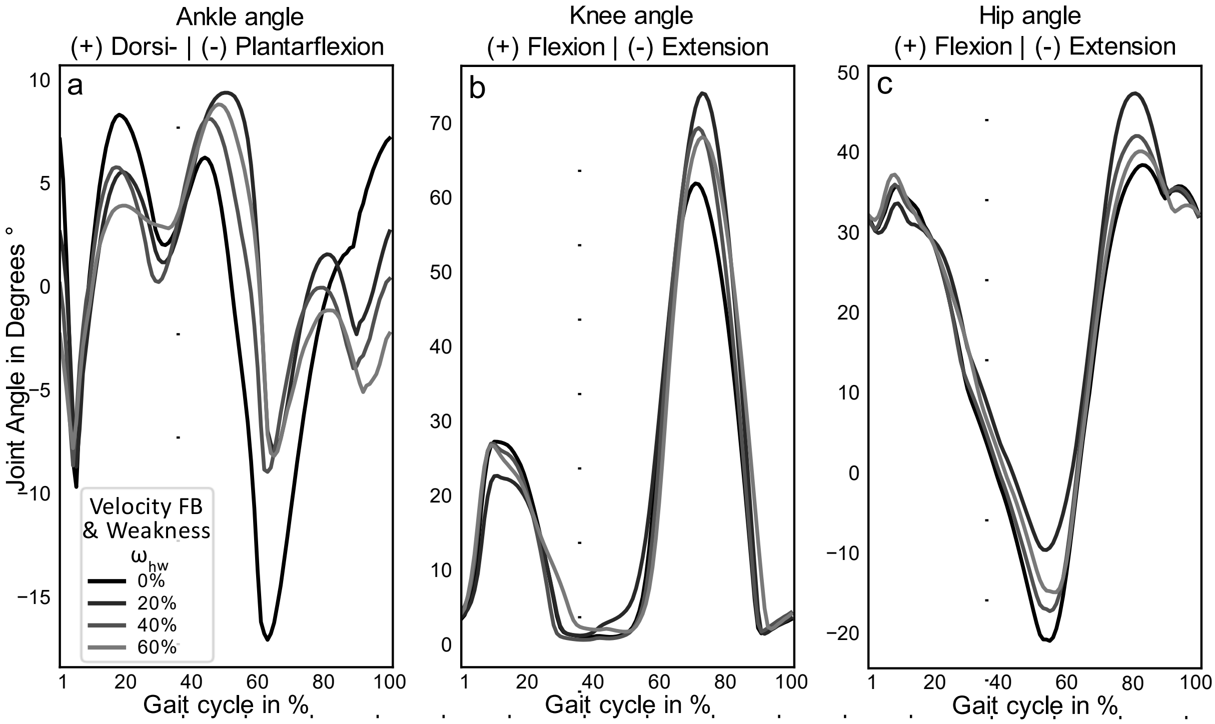

Figure 8: Flexion and extension angles of ankle, knee, and hip joints over the gait cycle in percent for velocity feedback gain and muscle weakness.

Figure 9. Simulated kinematics for toe-gait

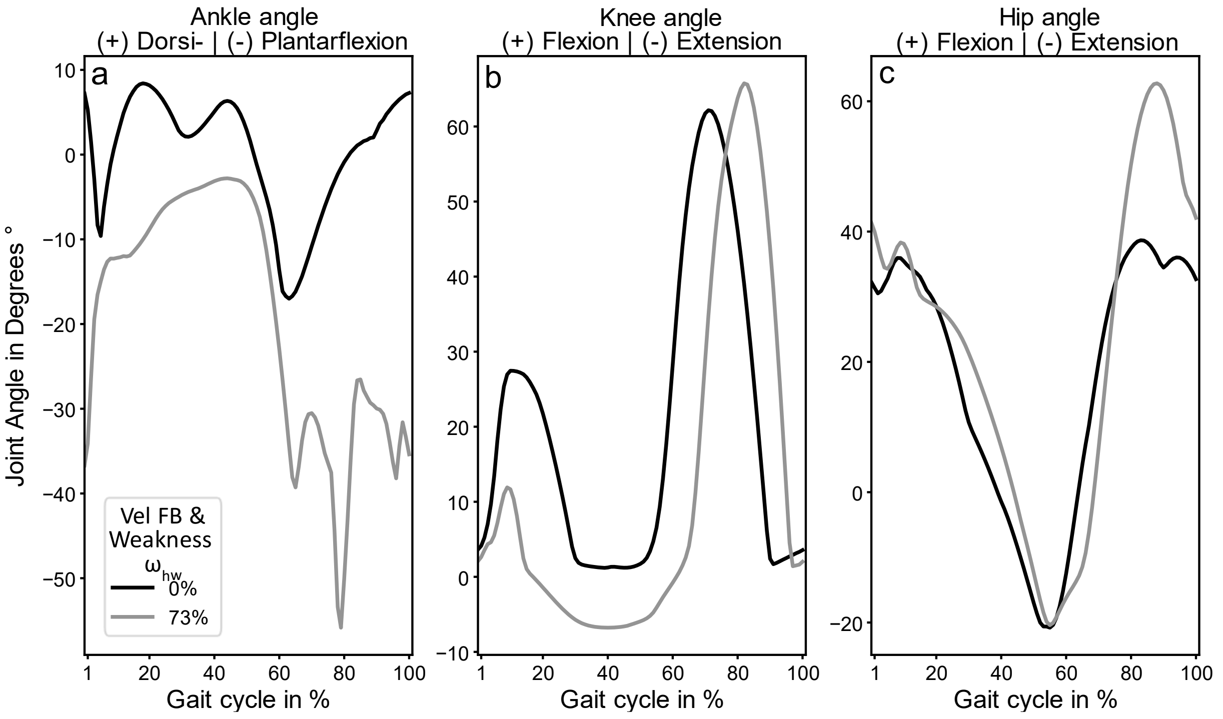

Figure 9: Flexion and extension angles of ankle, knee, and hip joints over the gait cycle in percent for velocity feedback gain and muscle weakness of $\omega_{hw} = 73\%$ *and* $\omega_{hw} = 0\%$ *for comparison. For this scenario, the optimization process produced a controller with stable toe gait with initial ball contact.*

Figure 10. Tibialis anterior activation and muscle spindle length feedback

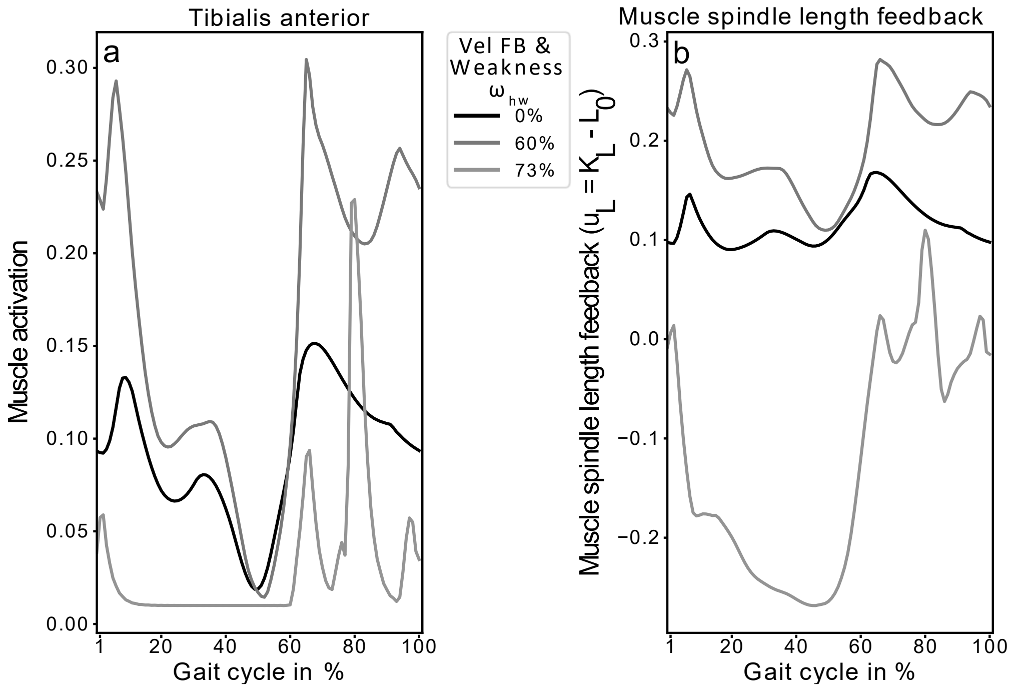

Figure 10: TA activation and muscle spindle length feedback over the gait cycle in percent for different levels of combined velocity feedback and muscle weakness ($\omega_{hw} = 0\%$, no alteration; $\omega_{hw} = 60\%$ heel strike gait; and $\omega_{hw} = 73\%$ toe-gait). For $\omega_{hw} = 73\%$ the muscle spindle length offset (L_0) is increased in comparison to other scenarios, which reduces TA activation and reciprocal inhibition of plantarflexor muscles.

Additional tables

Table 3: Experimental and simulation results in comparison

Mean results for gait features with standard deviation (STD) for experimental data taken from Laßmann et al. (2022). RoM ≡ Range of Motion, PF ≡ Plantarflexion, HS ≡ Heel strike, HGC ≡ Heel ground clearance. Asterisks indicate significance: ** if $p < 0.0056$ and *** if $p < 0.001$ in comparison to manifest SPG4 participants.

**Gait feature**	HC	prod SPG4	man SPG4
ankle RoM	33.7 $\pm$ 8.5***	31.5 $\pm$ 8.3	25.2 $\pm$ 6.9
min Plantarflexion	-20.8 $\pm$ 9.5***	-13.4 $\pm$ 10.5	-8.5 $\pm$ 7.9
ankle at HS	1.1 $\pm$ 7.2	5 $\pm$ 9.3	4.7 $\pm$ 6.3
ankle at max HGC	-18.6 $\pm$ 8.7***	-11.3 $\pm$ 10.6	-6.7 $\pm$ 8.5
knee RoM	60.2 $\pm$ 4.9***	58.2 $\pm$ 6.3**	47.1 $\pm$ 11.3
knee max angle	57.7$\pm$ 7.9	57 $\pm$ 7.2	47 $\pm$ 12.6
knee at HS	0.8 $\pm$ 6.8***	2.8 $\pm$ 5.7**	11.9 $\pm$ 7.9
gait speed [m/s]	1.36 $\pm$ 0.1***	1.28 $\pm$ 0.1	1.09 $\pm$ 0.2
stride length [cm]	146 $\pm$ 90	137 $\pm$ 11	116 $\pm$ 19

Table 4: Kinematic features for velocity feedback gain results over all iterations and correlation

Shown are all walking simulations for gradual increasing velocity feedback gains. In the bottom row spearman correlation coefficients for the respective features are shown. RoM ≡ Range of Motion, PF ≡ Plantarflexion, HS ≡ Heel strike, and HGC ≡ Heel ground clearance.

**Velocity Feedback ω_h**	Gait Speed	Stride Length	ankle RoM	Min PF	ankle at HS	ankle at max HGC	knee RoM	knee max angle	knee at HS
0%	1.20	1.48	25.43	-17.30	7.24	-14.70	60.99	62.27	3.59
6.7%	1.05	1.31	19.74	-11.67	3.99	-9.66	65.00	66.42	3.30
13.3%	1.10	1.36	19.60	-12.67	3.83	-8.95	63.13	64.98	3.50
20%	1.16	1.44	19.00	-11.45	2.31	-8.05	61.69	63.02	3.37
26.7%	1.07	1.35	16.25	-8.70	0.95	-5.75	62.29	63.34	3.11
33.3%	1.10	1.43	16.99	-9.70	-0.02	-7.38	62.72	62.88	4.17
40%	1.05	1.35	16.22	-8.35	-0.51	-5.11	61.92	63.36	3.79
46.7%	1.03	1.33	15.77	-7.81	-1.35	-3.93	62.94	64.12	3.82
53.3%	1.05	1.34	15.93	-4.29	-0.74	-2.24	64.35	65.51	9.73
60%	1.08	1.39	15.69	-6.17	-1.10	-3.27	62.22	62.70	11.50
66.7%	1.08	1.39	15.30	-7.78	-2.29	-3.97	62.79	63.44	9.57
73.3%	1.04	1.34	14.86	-5.82	-1.65	-1.98	60.87	63.10	12.66
80%	1.03	1.32	15.09	-7.35	-4.42	-3.52	62.97	63.95	4.26
86.7%	1.05	1.38	14.12	-5.04	-1.67	-2.32	61.03	61.73	13.01
93%	1.06	1.33	13.72	-4.78	-0.73	-2.32	65.52	67.12	18.35
**Correlation** Iteration 0 – 93%	rho=-0.53 p=0.04*	rho=-0.32 p=0.25	rho=-0.99 p<0.001***	rho=0.9 p<0.001***	rho=-0.87 p<0.001***	rho=0.88 p<0.001***	rho=0.06 p=0.82	rho=0.06 p=0.84	rho=0.88 p=0.001***

Table 5: Kinematic features for muscle weakness results over all iterations and correlation

Shown are all walking simulations for gradual increasing muscle weakness. In the bottom row spearman correlation coefficients for the respective features are shown. RoM ≡ Range of Motion, PF ≡ Plantarflexion, HS ≡ Heel strike, and HGC ≡ Heel ground clearance.

**Weakness ω_w**	Gait Speed	Stride Length	ankle RoM	Min PF	ankle at HS	ankle at max HGC	knee RoM	knee max angle	knee at HS
0%	1.20	1.48	25.43	-17.30	7.24	-14.70	60.99	62.27	3.59
6.7%	1.18	1.45	26.80	-15.50	8.58	-12.55	66.13	67.85	3.65
13.3%	1.10	1.35	24.89	-14.98	9.30	-10.24	68.55	69.89	3.54
20%	1.07	1.54	31.11	-19.05	8.83	-4.98	53.30	52.06	4.30
26.7%	0.96	1.18	21.00	-12.71	4.89	-9.04	72.13	73.37	3.42
33.3%	1.13	1.42	26.78	-17.66	8.32	-14.76	66.48	67.88	3.56
40%	1.14	1.33	22.84	-10.82	9.24	-3.64	74.37	75.43	2.77
46.7%	0.98	1.18	22.44	-12.97	8.49	-9.73	73.02	74.22	2.96
53.3%	0.96	1.45	28.33	-9.50	11.66	2.46	61.01	61.93	3.81
60%	1.10	1.33	24.72	-14.52	8.92	-12.91	68.46	70.41	3.27
66.7%	1.08	1.32	24.38	-13.80	10.63	-10.50	74.27	75.48	3.63
73.3%	1.07	1.28	25.85	-14.87	10.63	-12.78	72.90	74.42	3.14
80%	1.14	1.50	23.94	-12.59	11.34	-9.08	66.08	68.18	7.95
86.7%	0.99	1.30	26.83	-15.66	11.19	-12.50	68.20	69.68	3.58
93.3%	1.11	1.42	27.84	-14.48	13.42	-9.36	74.30	75.43	3.97
100%	1.11	1.51	28.65	-14.63	12.16	-11.72	65.65	67.87	7.63
**Correlation** Iteration 0-100%	rho=-0.19 p=0.47	rho=-0.09 p=0.74	rho=0.19 p=0.48	rho=0.33 p= 0.213	rho=0.8 p<0.001***	rho=0.06 p=0.81	rho= 0.25 p=0.35	rho=0.36 p=0.18	rho=0.26 p=0.33

Table 6: Kinematic features for velocity feedback gain and muscle weakness results over all iterations and correlation

Shown are all walking simulations for gradual increasing velocity feedback gain and muscle weakness. In the bottom row spearman correlation coefficients for the respective features are shown. RoM ≡ Range of Motion, PF ≡ Plantarflexion, HS ≡ Heel strike, and HGC ≡ Heel ground clearance, n.a. ≡ not applicable.

**Velocity FB + Weakness ω_{hw}**	Gait Speed	Stride Length	ankle RoM	Min PF	ankle at HS	ankle at max HGC	knee RoM	knee max angle	knee at HS
0%	1.20	1.48	25.43	-17.30	7.24	-14.70	60.99	62.27	3.59
6.7%	1.21	1.40	20.03	-13.32	1.59	-8.47	64.36	66.82	2.45
13.3%	1.06	1.33	21.45	-13.37	2.87	-10.72	63.23	66.52	3.50
20%	0.96	1.20	18.23	-8.67	2.56	-2.98	72.93	74.56	3.74
26.7%	1.12	1.36	18.60	-9.64	0.04	-5.96	65.40	67.29	3.40
33.3%	1.05	1.42	17.61	-9.27	3.45	-5.44	62.23	63.39	5.80
40%	1.03	1.33	17.26	-9.52	0.25	-3.89	68.82	69.94	4.55
46.7%	1.07	1.40	17.38	-8.30	0.10	-5.45	64.83	65.70	5.68
53.3%	1.06	1.30	16.74	-7.22	2.09	-1.84	73.45	74.70	4.34
60%	0.98	1.26	17.11	-8.40	-2.21	-4.88	66.54	69.26	4.33
66.7%	n.a.	n.a.	n.a.	n.a.	n.a.	n.a.	n.a.	n.a.	n.a.
73.3%	0.96	1.46	56.11	-70.33	-36.15	-31.53	72.69	66.19	2.06

**Correlation** Iteration 0 – 60%	rho=-0.52 p=0.13	rho=-0.26 p=0.47	rho=-0.98 p<0.001***	rho=0.96 p<0.001***	rho=-0.96 p<0.001***	rho=0.98 p<0.001***	rho=0.12 p=0.75	rho=0.01 p=0.99	rho=0.76 p=0.011

Table 7: Correlation of optimization parameters for each simulation experiment

Shown are spearman correlation coefficients for optimization parameters for each simulation experiment. TA ≡ tibialis anterior, SOL ≡ soleus, GAS ≡ gastrocnemius medialis, VAS ≡ vastus intermedius, K_L ≡ length feedback gain, L_0 ≡ length offset, K_F ≡ force feedback gain, and C_0 ≡ constant.

**Condition**	Optimization	Effort	TA[K_L]	TA[L_0]	TA-SOL[K_F]	SOL[K_F]	GAS[K_F]	VAS[K_F]	VAS[C_0]
Velocity feedback gain	rho=0.78, p=0.001***	rho=0.79, p<0.001***	rho=0.72, p=0.002**	rho=0.49, p=0.064	rho=-0.03, p=0.919	rho=-0.94, p<0.001***	rho=-0.94, p<0.001***	rho=-0.44, p=0.098	rho=-0.5, p=0.056
Muscle weakness	rho=-0.02, p=0.931	rho=-0.02, p=0.931	rho=0.41, p=0.113	rho=-0.01, p=0.957	rho=0.27, p=0.311	rho=0.54, p=0.031	rho=-0.23, p=0.399	rho=0.34, p=0.2	rho=0.0, p=1.0
Velocity feedback + muscle weakness	rho=0.63, p=0.05	rho=0.66, p=0.038	rho=0.47, p=0.174	rho=0.39, p=0.26	rho=0.48, p=0.162	rho=-0.98, p<0.001***	rho=-0.99, p<0.001***	rho=-0.48, p=0.162	rho=-0.84, p=0.002**
